# Biosynthesis of CDP-α‑d‑fucofuranose and CDP-β‑l‑6-deoxy-altrofuranose for the Capsular Polysaccharides of 

**DOI:** 10.1021/acs.biochem.5c00296

**Published:** 2025-08-08

**Authors:** Max Errickson Simons, Tamari Narindoshvili, Frank M. Raushel

**Affiliations:** † Department of Biochemistry & Biophysics, 2655 Texas A&M University, College Station, Texas 77842, United States; ‡ Department of Chemistry, Texas A&M University, College Station, Texas 77842, United States

## Abstract

is a Gram-negative human pathogen and is the most common cause of gastroenteritis in the United States and Europe. expresses a capsular polysaccharide (CPS) that enables the evasion of the host immune response and adherence to host epithelial cells. The various subspecies (serotypes) of are distinguished by their unique CPS repeating units. The repeating trisaccharide in the HS:41 serotype was previously found to contain l-arabinofuranose, 6-deoxy-d-*altro*-heptofuranose, and a third sugar, which is either d-fucofuranose or 6-deoxy-l-altrofuranose. Genome neighborhood and sequence similarity networks were employed to identify five candidate genes for the biosynthesis of d-fucofuranose and 6-deoxy-l-altrofuranose. Here, it was demonstrated that the biosynthetic pathways for both sugars are initiated by the formation of CDP-d-glucose from CTP and d-glucose-1-phosphate as catalyzed by HS41.21. This product is dehydrated by an NAD^+^-dependent 4,6-dehydratase (HS41.20) that produces CDP-4-keto-6-deoxy-d-glucose. The third enzyme (HS41.19) was shown to catalyze the inversion of stereochemistry at C3 and C5 using CDP-4-keto-6-deoxy-d-glucose as the substrate. The fourth enzyme (HS41.18) is an NADPH-dependent C4-reductase that catalyzes the formation of CDP-d-fucopyranose from CDP-4-keto-6-deoxy-d-glucose in the absence of the 3,5-epimerase but catalyzes the formation of CDP-6-deoxy-l-altropyranose in the presence of the epimerase. The last enzyme (HS41.17) in the pathway was shown to catalyze the FADH_2_-dependent interconversion of CDP-d-fucopyranose and CDP-6-deoxy-l-altropyranose to CDP-d-fucofuranose and CDP-6-deoxy-l-altrofuranose, respectively. The overall synthesis of the two possible products is governed by the catalytic activity of the epimerase, since the C4-reductase and pyranose–furanose mutase are not affected by the stereochemistry at C5.

## Graphical abstract

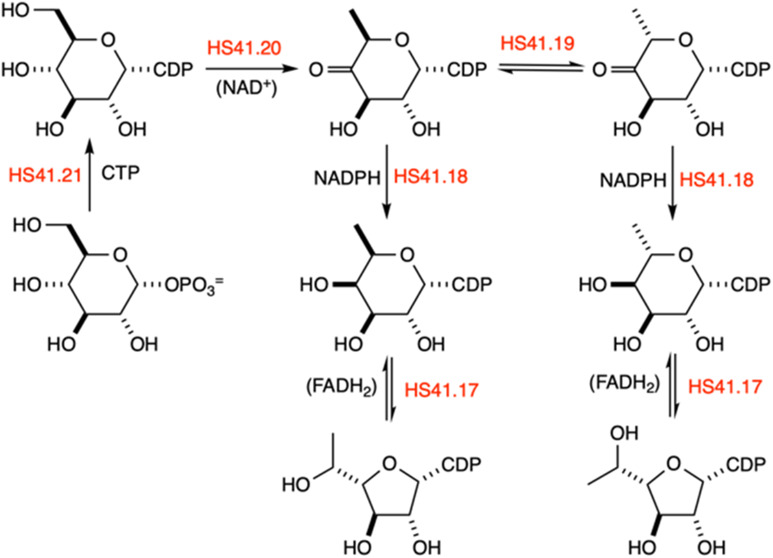

## Introduction

 is a Gram-negative pathogenic bacterium that is responsible for most of the gastroenteritis cases in Europe and the United States.^1^ The bacterium is commensal in the digestive tracts of poultry, and individuals infected by are at risk of autoimmune disorders such as Guillain–Barré Syndrome.^2^ Currently, there are no FDA-approved vaccines.^3,4^ synthesizes a capsular polysaccharide (CPS) that is subsequently exported to the cell surface, which helps the bacterium evade the host immune response.^5,6^ The CPS is a repeating polysaccharide of 2–4 different monosaccharides connected to one another via glycosidic bonds. Attempts to formulate the CPS as the basis for a bioconjugate vaccine have shown promise; however, has at least 33 known serotypes, and each serotype has a distinct CPS.^3,7^

The chemical composition of the CPS from serotype HS:41 of (strain 176.83) has been determined, and the chemical structures of the two recurring motifs are shown in Figure 1.^8^ The minor trisaccharide repeating unit consists of l-arabinose, 6-deoxy-d-*altro*-heptose, and d-fucose (shown as compound **1**), whereas the major repeating trisaccharide consists of l-arabinose, 6-deoxy-d-*altro*-heptose, and 6-deoxy-l-altrose (shown as compound **2**). These two structures differ only in the stereochemistry at C5 of the d-fucose/6-deoxy-l-altrose moiety. As reported, the ratio of 6-deoxy-l-altrose to d-fucose is ∼3:1.^8^ However, it could not be determined whether an individual CPS strand contained a mixture of 6-deoxy-l-altrose and d-fucose or whether the analyzed sample contained a mixture of CPS strands that contained either 6-deoxy-l-altrose or d-fucose.^8^ The gene cluster for the biosynthesis of CPS from serotype HS:41 is presented in Figure 2. It has been demonstrated previously that the eight genes denoted here as HS41.07, HS41.08, and HS41.10–HS41.15 are required for the biosynthesis of GDP-6-deoxy-d-*altro*-heptose and that the genes denoted here as HS41.24–HS41.27 are required for the biosynthesis of UDP-l-arabinose.^9–13^ However, it is not currently understood how either d-fucose or 6-deoxy-l-altrose is biosynthesized or how two different sugars can be incorporated into the same positional space within the polysaccharide.

**Figure 1:**
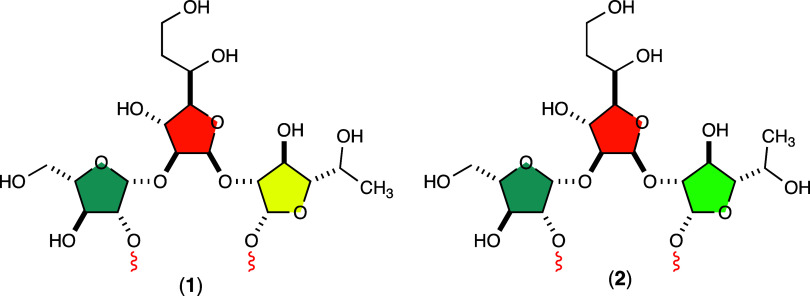
Repeating structures in the capsular polysaccharide of serotype HS:41 of . In structure **1**, the repeating trisaccharide consists of l-arabinose (highlighted in blue), 6-deoxy-d-*altro*-heptose (highlighted in orange), and d-fucose (highlighted in yellow). In structure **2**, the repeating trisaccharide consists of l-arabinose, 6-deoxy-d-*altro*-heptose, and 6-deoxy-l-altrose (highlighted in green).

**Figure 2:**
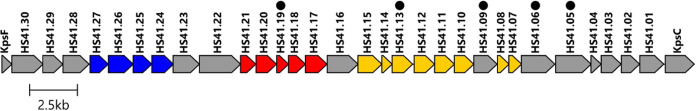
CPS gene cluster for the biosynthesis of the capsular polysaccharide of the HS:41 serotype of strain 176.83. The genes required for the biosynthesis of GDP-6-deoxy-d-*altro*-heptose are highlighted in yellow, and those required for the biosynthesis of UDP-l-arabinose are highlighted in blue. The proposed genes for the biosynthesis of the activated forms of d-fucose and/or 6-deoxy-l-altrose are highlighted in red. The black dots indicate the presence of a poly-G/C tract within the DNA.

In the present investigation, we have focused on the elucidation of the biosynthetic pathways for the construction of the activated forms of d-fucose and 6-deoxy-l-altrose. In the absence of any current experimental evidence, it could be imagined that one of the two possible NDP-sugars could be biosynthesized and subsequently incorporated into the growing polysaccharide chain, and then an additional enzyme would facilitate the isomerization of the stereochemistry at C5. Alternatively, it could be imagined that both NDP-sugars are biosynthesized and then randomly incorporated into the growing polysaccharide chain by using the same or separate glycosyl transferases. Based upon the provisional annotations, as identified in UniProt, for the enzymes of unknown function contained within the gene cluster for the biosynthesis of the CPS in the HS:41 serotype of , the most likely set of genes for the construction of NDP-d-fucose and/or NDP-6-deoxy-l-altrose appears to be HS41.17 – HS41.21.

HS41.21 (UniProt ID: Q5M6T0) is provisionally annotated as a sugar-1-phosphate cytidylyltransferase, whereas HS41.20 (UniProt ID: Q5M6T1) is annotated as a nucleotidyl dehydratase. HS41.19 (UniProt ID: Q5M6T2) is suggested to catalyze the epimerization of dTDP-4-dehydrorhamnose, while HS41.18 (UniProt ID: Q5M6T3) is also annotated as a nucleotidyl-sugar dehydratase. Finally, HS41.17 (UniProt ID: Q5M6T4) is likely to be a nucleotidyl-sugar pyranose/furanose mutase. Based on these highly provisional annotations, we propose the pathway illustrated in Figure 3 for the biosynthesis of the activated forms of d-fucose and 6-deoxy-l-altrose for incorporation into the capsular polysaccharide of the HS:41 serotype of .

**Figure 3:**
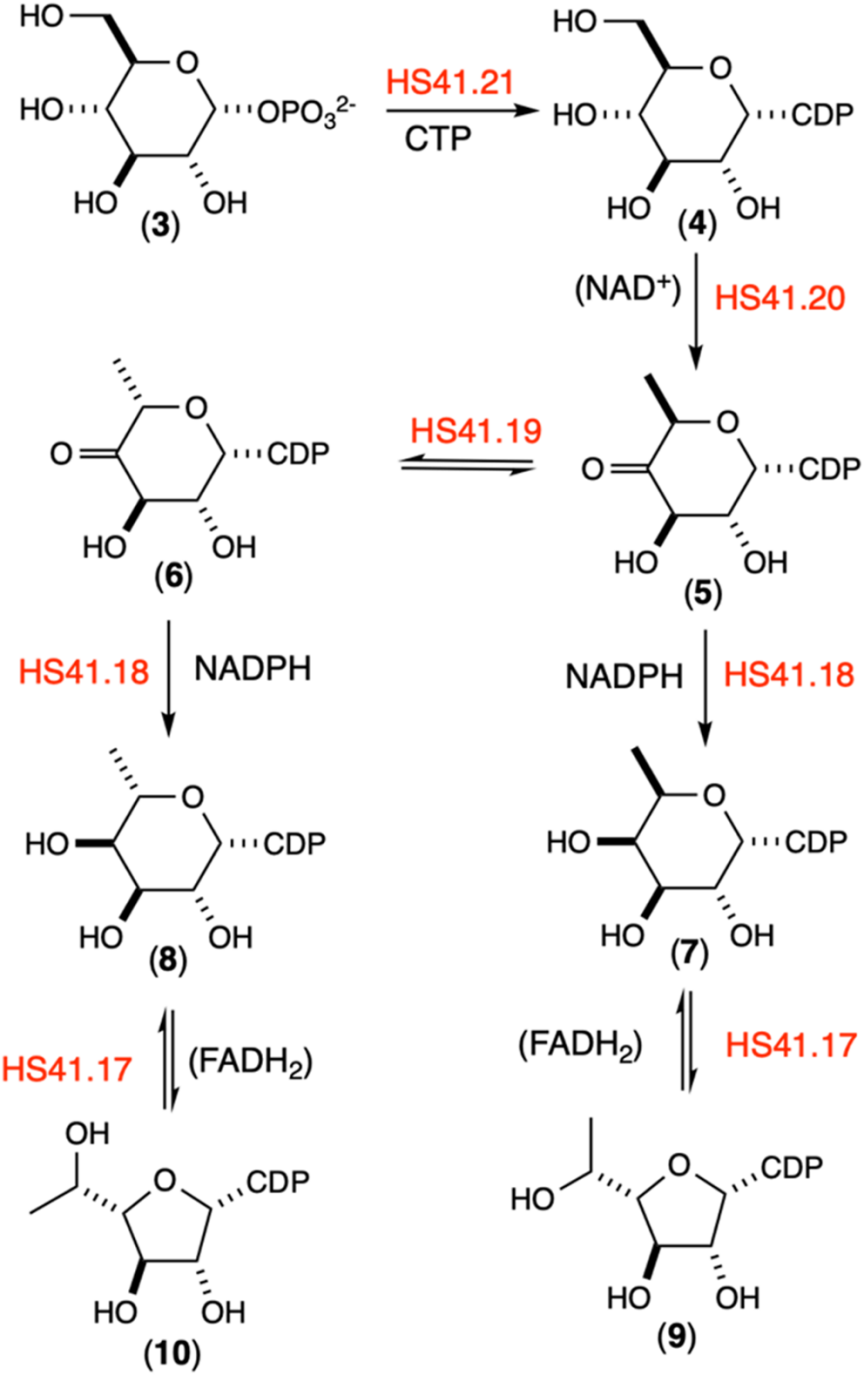
Proposed biosynthetic pathway for construction of CDP-α-d-fucofuranose (**9**) and CDP-β-l-6-deoxy-altrofuranose (**10**) starting from d-glucose-1-phosphate (**3**). Additional details are available in the text.

In the proposed biosynthetic pathway, HS41.21 catalyzes the reaction of d-glucose-1-phosphate (**3**) with CTP to form CDP-d-glucose (**4**) and PP_i_. In the next step, HS41.20 catalyzes the NAD^+^-dependent oxidation/dehydration/reduction of CDP-d-glucose (**4**) to CDP-6-deoxy-4-keto-d-glucose (**5**). The formation of the carbonyl group at C4 of compound **5** then facilitates the isomerization of C5 and formation of CDP-4-keto-6-deoxy-l-altrose (**6**). In the penultimate step, HS41.18 catalyzes the reduction of the carbonyl groups of compounds **5** and **6** to CDP-d-fucose (**7**) and CDP-6-deoxy-l-altrose (**8**), respectively. In the final step, HS41.17 catalyzes the FADH_2_-dependent conversion of the pyranose configurations of **7** and **8** to the corresponding furanose forms, **9** and **10**. The proposed biosynthetic pathway postulates that two enzymes (HS41.18 and HS41.17) catalyze their respective transformations irrespective of the stereochemistry at C5, a requirement that is necessitated by the lack of other candidate enzymes contained within the gene cluster.

## Materials and Methods

### Materials

Isopropyl β-d-1-thiogalactose (IPTG) and Luria Broth were purchased from Research Products International. NADPH, CTP, FAD, ATP, UTP, dTTP, HEPES, imidazole, α-d-glucose-1-phosphate, α-d-galactose-1-phosphate, and dithiothreitol (DTT) were purchased from Sigma-Aldrich, in addition to yeast pyrophosphatase and rabbit muscle phosphoglucomutase (PGM). The 5 mL Ni-NTA HisTrap columns and the Vivaspin 20 (10 kDa molecular weight cutoff) protein concentrators were obtained from GE Healthcare and Cytiva, respectively. The 5 mL HiTrap Q anion exchange columns and Carbopac Dionex PA1 columns were purchased from GE Healthcare. [^18^O]-water (97%) was purchased from Medical Isotopes Inc. The chemical syntheses of CDP-α-d-fucofuranose (**9**), CDP-β-6-deoxy-l-altrofuranose (**10**) and the chemo-enzymatic synthesis of the oxygen-18 labeled UDP-d-fucose (**7x**) are described in the Supporting Information.

### Isolation of Cytidylyltransferase HS41.21

The gene for the cytidylyltransferase HS41.21 (UniProt ID: Q5M6T0) was chemically synthesized by Twist Bioscience into a pET29 expression vector to produce a protein with a polyhistidine purification tag at the C-terminus. The plasmid was transformed into BL21­(DE3), and the cells were grown on plates supplemented with 50 μg/mL kanamycin. A starter culture was used to inoculate the LB medium supplemented with 50 μg/mL kanamycin and incubated at 37 °C until reaching an OD of ∼0.6. The cells were induced with 0.2 mM IPTG, grown overnight at 16 °C, and then centrifuged at 15,000 rcf for 15 min. The cells were resuspended in Buffer A (20 mM imidazole, 50 mM HEPES/K^+^, 250 mM KCl, pH 8.5), followed by sonication. The lysate was centrifuged at 15,000 rcf for 30 min, filtered (Whatman 0.45 μm nitrocellulose), injected onto a Ni-NTA column, and then eluted from the column with a gradient of Buffer B (500 mM imidazole, 50 mM HEPES/K^+^, 250 mM KCl, pH 8.5). The purified protein was concentrated with a Vivaspin 20 (10 kDa MWCO) filter to 0.4 mg/mL, frozen in liquid nitrogen, and then stored at −80 °C. Approximately 1.1 mg of purified enzyme was obtained per liter of cell culture. The molecular weight of the purified protein was 32.9 kDa with a computed molar extinction coefficient of 64,000 M^–1^ cm^–1^ at 280 nm.^14^

### Isolation of 4,6-Dehydratase HS41.20

The gene for 4,6-dehydratase HS:41.20 (UniProt ID: Q5M6T1) was chemically synthesized by Twist Bioscience into a pET28 expression vector to produce an enzyme with an N-terminal polyhistidine purification tag. The plasmid was transformed into BL21­(DE3) cells on plates supplemented with 50 μg/mL kanamycin. The LB medium, containing 50 μg/mL kanamycin, was inoculated with a starter culture, grown to an OD of ∼0.6, and then induced with 1.0 mM IPTG. The cells were incubated overnight at 21 °C, pelleted at 15,000 rcf for 15 min, and then resuspended in Buffer A, followed by sonication. The lysate was centrifuged at 15,000 rcf for 30 min, filtered, injected onto a Ni-NTA column, and then eluted with a gradient of Buffer B. The protein was concentrated to 0.6 mg/mL, frozen in liquid nitrogen, and stored at −80 °C. A typical purification yielded 2.4 mg of enzyme per liter of cell culture. The isolated enzyme HS41.20 has a molecular weight of 42.9 kDa and a computed extinction coefficient of 73,500 M^–1^ cm^–1^ at 280 nm.^14^

The ultraviolet–visible (UV–vis) spectrum of the purified enzyme exhibited an absorbance maximum at 342 nm, indicating the presence of either NADH or NADPH bound to the active site. To determine the identity of the bound cofactor, a 1.0 mL sample of the purified enzyme (10 μM) was denatured by heating for 2 min at 100 °C. The precipitated protein was removed by centrifugation, and the supernatant solution was loaded onto a HiTrap Q anion exchange column, followed by elution using a gradient of 0–0.5 M ammonium bicarbonate (pH 7.5) while monitoring the effluent at 255 nm. To confirm the identity and stoichiometry of the bound cofactor, a fixed concentration of NADPH (10 μM) was added to the enzyme sample prior to heat denaturation, and the reaction mixture was then characterized via the same procedure.

### Isolation of 3,5-Epimerase HS41.19

The gene for HS41.19 (UniProt ID: Q5M6T2) was chemically synthesized by Twist Bioscience into a pET28 expression vector for the production of an enzyme with a polyhistidine purification tag at the N-terminus. The plasmid was transformed into BL21­(DE3) cells and grown on plates supplemented with 50 μg/mL of kanamycin. The LB growth medium, supplemented with 50 μg/mL kanamycin, was inoculated with a starter culture and grown at 37 °C until reaching an OD of ∼0.6. Protein production was induced with 0.5 mM IPTG, and the cells were grown overnight at 25 °C. The cells were isolated by centrifugation at 15,000 rcf for 15 min and then resuspended in Buffer A. After sonication, the lysate was centrifuged at 15,000 rcf for 30 min, filtered, and then injected onto the Ni-NTA column. The enzyme was eluted from the column with a gradient of Buffer B, and then concentrated to 0.7 mg/mL before being frozen and stored at −80 °C. The yield of purified protein was ∼1.4 mg per liter of cell culture. The isolated enzyme has a molecular weight of 26.6 kDa and a computed molar extinction coefficient of 23,600 M^–1^ cm^–1^ at 280 nm.^14^

### Isolation of the C4-Reductase HS41.18

The gene for HS41.18 (UniProt ID: Q5M6T3) was chemically synthesized by Twist Bioscience into a pET28 expression vector for the production of protein with a polyhistidine purification tag at the N-terminus. The plasmid was transformed into BL21­(DE3) cells on plates supplemented with 50 μg/mL kanamycin. LB medium supplemented with 50 μg/mL kanamycin was inoculated with a starter culture and incubated until reaching an OD of ∼0.6 before being induced with 1.0 mM IPTG and allowed to grow overnight at 16 °C. The cells were centrifuged at 15,000 rcf for 15 min and then resuspended in Buffer A. After sonication, the lysate was centrifuged at 15,000 rcf for 30 min, filtered, and then injected onto the Ni-NTA column. The enzyme was eluted from the column using a gradient of Buffer B and then concentrated to 1.4 mg/mL. The isolated enzyme was frozen in liquid nitrogen and stored at −80 °C. The yield of the purified enzyme was 3.3 mg per liter of cell culture. The isolated enzyme has a molecular weight of 36.6 kDa and a computed molar extinction coefficient of 39,000 M^–1^ cm^–1^ at 280 nm.^14^

### Isolation of Pyranose/Furanose Mutase HS41.17

The gene for HS:41.17 (UniProt ID: Q5M6T4) was chemically synthesized by Twist Bioscience into a pET28 expression vector for the expression of protein with a polyhistidine purification tag at the N-terminus. Arctic express cells were transformed with the plasmid on plates containing 20 μg/mL gentamycin and 50 μg/mL kanamycin. The LB medium, supplemented with 1.0 mM DTT, 20 μg/mL gentamycin, and 50 μg/mL kanamycin, was inoculated with the transformed cells and then incubated at 37 °C until reaching an OD of ∼0.6. Gene expression was induced with 0.5 mM IPTG and then grown for 24 h at 14 °C. Cells were pelleted at 15,000 rcf for 15 min, resuspended in Buffer A, and then sonicated. The lysate was supplemented with 0.2% Triton X, centrifuged at 15,000 rcf for 30 min, filtered, and then injected onto a Ni-NTA column. The enzyme was eluted from the column with a gradient of Buffer B and then concentrated to 0.5 mg/mL. The isolated enzyme was frozen in liquid nitrogen and stored at −80 °C. The yield of purified protein was 1.3 mg/L of cell culture. The enzyme has a molecular weight of 46.7 kDa and a computed molar extinction coefficient of 74,200 M^–1^ cm^–1^ at 280 nm.^14^ The amino acid sequences of the five purified proteins are provided in Figure S1, and representative SDS gels of the purified proteins are shown in Figure S2.

The purified enzyme was yellow in color. To confirm the presence of FAD, a 1.0 mL sample of enzyme (40 μM) was heat-denatured for 2 min at 100 °C and then centrifuged to remove the precipitated protein. The supernatant solution was injected onto a 5 mL HiTrap Q anion exchange column and then eluted with a linear gradient of 0–0.5 M ammonium bicarbonate. Fractions that absorbed at 255 nm were pooled, lyophilized, and then rehydrated in 50 mM HEPES, pH 7.5.

### Characterization of Glucose-1-phosphate Cytidylyltransferase

The reaction catalyzed by HS41.21 was initially characterized by incubation of the purified enzyme (1.0 μM), 8.0 mM CTP, 6.0 mM Glc1P (**3**), 7.0 mM MgCl_2_, and 60 nM pyrophosphatase in a volume of 1.0 mL for 18 h at 25 °C in 50 mM HEPES/K^+^ buffer (pH 8.5). The reaction product was isolated by chromatography using a 5 mL HiTrap Q anion exchange column by monitoring the absorbance at 255 nm and elution with a linear gradient of 0–500 mM ammonium bicarbonate (pH 7.5). The isolated product (**4**) was lyophilized to dryness and characterized by ^1^H NMR spectroscopy and ESI-mass spectrometry.

The values of *k*_cat_ and *k*_cat_/*K*_m_ for the reaction catalyzed by Glc1P cytidylyltransferase were determined at 25 °C by measuring the rate of formation of CDP-Glc (**4**) using anion exchange chromatography to separate the substrate CTP from the product. The reaction mixtures contained 50 mM HEPES/K^+^ (pH 7.5), 2.0 mM CTP, 3.0 mM MgCl_2_, 60 nM pyrophosphatase, and 0.075 to 2.0 mM Glc1P (**3**). The reactions were initiated by the addition of 0.36 μM enzyme and subsequently quenched at various times by heating at 100 °C for 2 min. The kinetic constants were obtained by a fit of the data to the Michaelis–Menten equation.

### Characterization of CDP-Glc-4,6-Dehydratase

The reaction catalyzed by HS41.20 was determined by a combination of ESI-mass spectrometry and ^1^H NMR spectroscopy. For the ESI-MS experiments, the enzyme (1.0 μM) was incubated with 3.0 mM CDP-Glc (**4**) in 50 mM NH_4_HCO_3_ buffer (pH 7.5) at 25 °C for 3 h, and an identical experiment was conducted in D_2_O (90%). For the ^1^H NMR experiments, 6.0 mM CDP-Glc (**4**) was incubated with 1.0 μM enzyme in 50 mM potassium phosphate buffer (pD 7.5) in D_2_O (95%) at 25 °C for 7 h.

The kinetic constants for the reaction catalyzed by CDP-Glc-4,6-dehydratase (HS41.20) were determined using a coupled enzyme assay that utilized an excess of the C4-reductase (HS41.18) in the presence of NADPH to reduce the product (**5**) to CDP-d-Fuc (**7**). The assays were conducted in a 1.0 mL cuvette and contained 50 mM HEPES/K^+^ (pH 7.5), 0.16 mM NADPH, 1.25 μM C4-reductase (HS:41.18), 120 nM CDP-Glc-4,6-dehydratase (HS41.20), and variable amounts of CDP-Glc (3.0–60 μM). The reactions were conducted at 25 °C and monitored for a change in absorbance at 340 nm. The data were fit to the Michaelis–Menten equation to obtain the values of *k*_cat_ and *k*_cat_/*K*_m_.

### Characterization of CDP-4-keto-6-deoxy-d-glucose-3,5-epimerase

The reaction catalyzed by 3,5-epimerase (HS41.19) was initially analyzed by ^1^H NMR spectroscopy and ESI-mass spectrometry. For the ESI-MS experiments, 0.5 mM CDP-4-keto-6-deoxy-glucose (**5**) was incubated with 1.5 μM of the 3,5-epimerase in 50 mM NH_4_HCO_3_ for 60 min at 25 °C in a volume of 200 μL in 90% D_2_O. For the ^1^H NMR experiments, 2.0 mM CDP-Glc in 50 mM NH_4_HCO_3_ was incubated with 0.25 μM CDP-Glc-4,6-dehydratase and 3.0 μM of the 3,5-epimerase for 18 h at 25 °C in a solution of 90% D_2_O.

The rate of the 3,5-epimerase-catalyzed reaction was determined by ESI-mass spectrometry. Solutions of 0.5–1.0 mM CDP-glucose (**4**) were first dehydrated in either 100% H_2_O or 90% D_2_O using 0.7 μM 4,6-dehydratase in 50 mM ammonium bicarbonate buffer, pH 7.5, overnight, and then the 4,6-dehydratase was removed by filtration. The samples were then incubated with 1.5 μM of the 3,5-epimerase in either 10 or 90% D_2_O, and aliquots of the mixtures were taken as a function of time before heating at 100 °C for 1 min. The products were analyzed by ESI-MS, and the hydrogen–deuterium exchange rates were determined by a fit of the data to a single exponential equation.

### Characterization of the C4-Reductase

The products of the C4-reductase-catalyzed reaction were characterized by ESI-mass spectrometry and ^1^H NMR spectroscopy. For these experiments, a solution of 6.0 mM CDP-Glc (**4**) in 50 mM HEPES/K^+^, pH 7.5, was first dehydrated to compound **5** using 1.0 μM of the 4,6-dehydratase in a reaction volume of 500 μL at 25 °C. After the dehydration reaction was complete, NADPH and the C4-reductase were added to a concentration of 5.0 mM and 0.5 μM, respectively, in a final volume of 1.0 mL to synthesize CDP-d-Fuc (**7**). The same transformation was conducted in 50 mM phosphate buffer (pH 7.5) in 90% D_2_O to synthesize the deuterated product at C5. Identical reactions were conducted in the presence of the 3,5-epimerase (2.0 μM) to synthesize a mixture of CDP-d-fucose (**7**) and CDP-6-deoxy-l-altrose (**8**). The samples were applied to a Carbopac Dionex PA1 column and eluted with a linear gradient of 0–0.5 M ammonium acetate (pH 7.5), and the products monitored at 255 nm.

To determine the effect of the 3,5-epimerase on the product distribution of CDP-d-fucose (**7**) and CDP-6-deoxy-l-altrose (**8**), reduction of compound **5** was conducted using different ratios of the C4-reductase and 3,5-epimerase in the presence of NADPH. The experiments were conducted in 50 mM P_i_ buffer in D_2_O (pD 7.5) with 3.0 mM NADPH and 2.0 mM CDP-4-keto-6-deoxy-glucose (**5**) in the presence of the C4-reductase (1.5 μM) using either 0, 55 nM, or 1.5 μM of the 3,5-epimerase in a volume of 500 μL. The reactions were allowed to incubate at 25 °C for 24 h. The samples were applied to a Carbopac Dionex PA1 column and eluted with a linear gradient of 0–0.5 M ammonium acetate (pH 7.5), and the products were monitored at 255 nm.

The values of *k*_cat_ and *k*_cat_/*K*_m_ for the reduction of CDP-4-keto-6-deoxy-glucose (**5**) by the C4-reductase to CDP-d-Fuc (**7**) were determined in the presence of 0.16 mM NADPH in 50 mM HEPES/K^+^, pH 7.5, at 25 °C. The substrate (**5**) was made *in situ* by the addition of the 4,6-dehydratase to a 1.0 mM solution of CDP-Glc (**4**) and then diluted to the desired concentration. The reaction was initiated by the addition of the C4-reductase (45 nM), and the change in absorbance was monitored at 340 nm. An identical set of measurements were conducted in the presence of the 3,5-epimerase (1.0 μM). The final substrate concentration (based on the initial concentration of CDP-Glc) ranged from 2.5 to 250 μM.

### Characterization of the Pyranose/Furanose Mutase

The reaction catalyzed by the putative pyranose/furanose mutase (HS41.17) was initially addressed by incubation of 1.0 mM CDP-d-fucofuranose (**9**) with 5.0 nM HS41.17, 25 mM KCl, 20 mM sodium dithionite, and 50 mM phosphate/K^+^ buffer (pH 7.0) in a volume of 0.5 mL. After 2 h, the reaction was quenched by heating the sample at 100 °C for 2 min. After centrifugation, an aliquot was injected onto a 1 mL Carbopac PA1 column, and the product CDP-α-d-fucopyranose (**7**) was separated from CDP-α-d-fucofuranose (**9**) using a linear gradient of 0–2.0 M ammonium acetate. Identical experiments were also conducted using chemically synthesized CDP-6-deoxy-altrofuranose (**10**) and its conversion to CDP-l-6-deoxy-altropyranose (**8**) and the reverse reaction starting with **8**. The rate of conversion of 1.0 mM furanose **9** to the pyranose **7** was determined by the removal of aliquots as a function of time and determining the ratio of **7** and **9** after separation using the Carbopac PA1 column while monitoring the effluent at 255 nm. These reactions were conducted at pH 7.0 in 50 mM P_i_ buffer using 5.0 nM mutase in the presence of 25 mM KCl and 20 mM sodium dithionite.

The catalytic properties of HS41.17 were further interrogated by monitoring the positional isotope exchange within [1-^18^O]-CDP-d-fucose (**7x**).^13,15^ In these experiments, a 2.5 mM solution of **7x** was incubated in a volume of 0.5 mL with 0.75 μM HS41.17, 20 mM sodium dithionite, 25 mM KCl, and 25 mM phosphate/K+ buffer (pH 7.5) for 3 h. The potential reaction products (**7y** and **7z**) were analyzed by ^31^P NMR spectroscopy.

## Results and Discussion

### Glucose-1-phosphate Cytidylyltransferase

The gene for the putative Glc1P cytidylyltransferase was expressed in and purified to homogeneity. The reaction product from the incubation of the enzyme with MgCTP and Glc1P was isolated via anion exchange chromatography, and the proposed structure (compound **4**) was confirmed by NMR spectroscopy and ESI-mass spectrometry (Figures S3 and S4). The observed *m*/*z* for the [M-H]^−^ anion is 564.06, which is fully consistent with the theoretical value of 565.06 for CDP-Glc (**4**). Substitution of CTP with either UTP, ATP, or dTTP failed to support catalytic activity, as did the substitution of Gal1P for Glc1P. The rate of the reaction was determined by measuring the formation of CDP-Glc (**4**) as a function of time by anion exchange chromatography at a fixed initial concentration of MgCTP and variable concentrations of Glc1P (Figure S5). The measured values of *k*_cat_ and *k*_cat_/K_Glc1P_ are 0.47 (±0.02) s^–1^ and 1.8 (±0.2) × 10^3^ M^–1^ s^–1^, respectively. These experiments confirm that HS41.21 catalyzes the formation of CDP-Glc (**4**) from MgCTP and Glc1P (**3**).

### CDP-Glc-4,6-dehydratase

The dehydratase was purified to homogeneity, and the UV–vis spectrum showed an additional absorbance maximum at 342 nm, indicating the presence of either NADPH or NADH bound to the active site (Figure S6). To determine the identity of the bound cofactor(s), the purified enzyme was heat-denatured, and the supernatant solution, after centrifugation, was applied to an anion exchange column. The elution profile showed the presence of two compounds (Figure 4b) with retention times that are identical to those of NAD^+^ and NADH (Figure 4a). To estimate the stoichiometry of the two bound cofactors (NAD^+^ and NADH), the denaturation and chromatography experiments were repeated with an enzyme sample that contained a known amount of added NADPH (Figure 4c). Quantitation of the areas in the elution profile for each compound with the initial concentration of the dehydratase indicates that 27% of the active sites are bound with NAD^+^ and 39% are bound with NADPH. A third compound was also isolated after chromatography, lyophilized, reconstituted in NH_4_HCO_3_, and subjected to ESI-mass spectrometry. The *m*/*z* for the [M-H]^−^ anion of 402.01 amu is consistent with the theoretical value of CDP. The presence of NAD^+^ in the active site of the 4,6-dehydratase is required to facilitate the oxidation of C4, dehydration of C6, and reduction of the resulting double bond between C5 and C6 (Scheme S1).^16^

**Figure 4:**
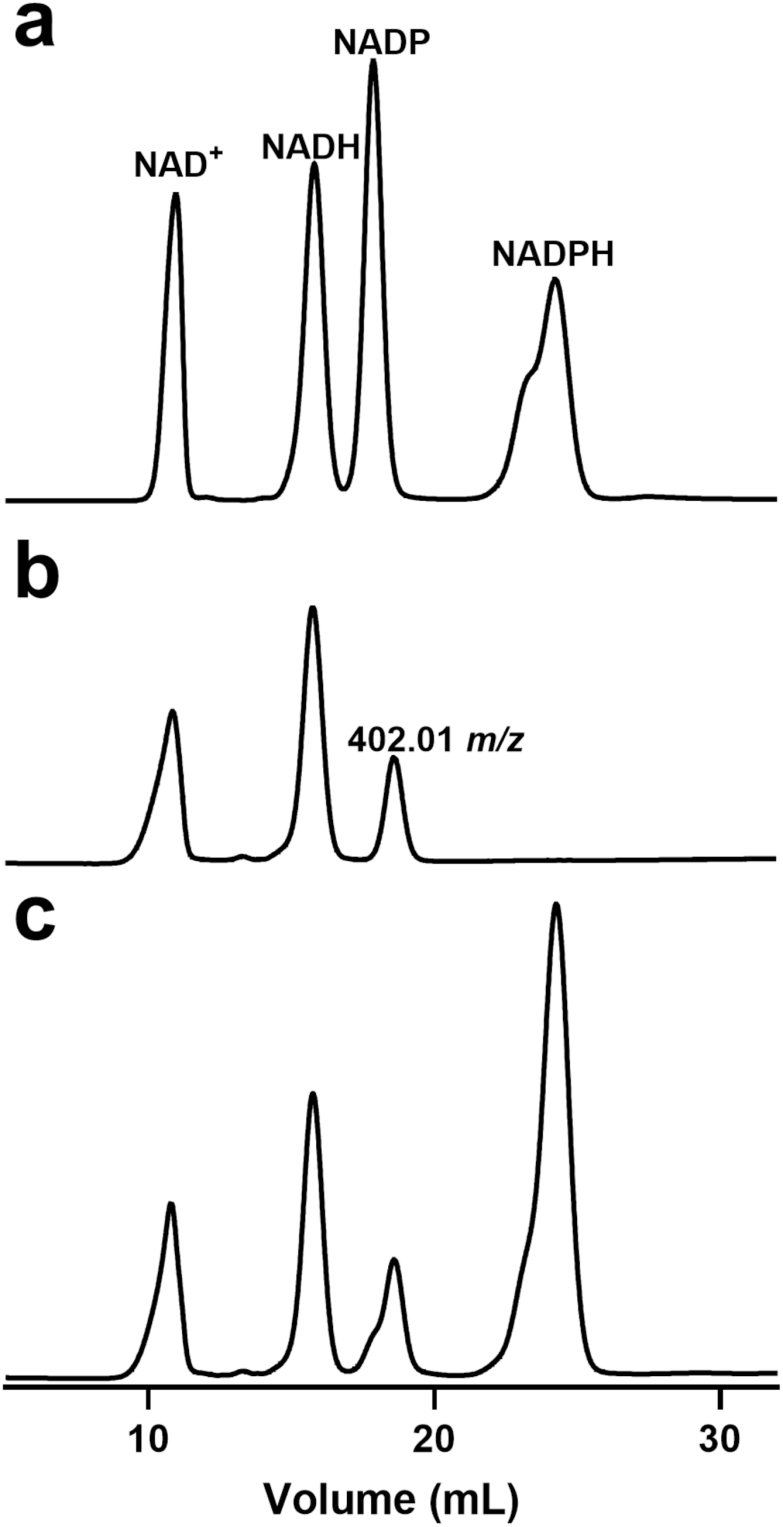
Identification of bound ligands to the as-purified form of HS41.20 after heat denaturation and anion exchange chromatography. (a) Control sample containing equal concentrations of NAD^+^, NADH, NADP^+^, and NADPH. (b) Compounds isolated after the heat denaturation of HS41.20. The third compound was identified as CDP based on the observed *m*/*z* of 402.01 for the [M-H^+^]^−^ anion. (c) Compounds isolated after heat denaturation of HS41.20, plus 10 μM NADPH added as an internal standard. Additional details are given in the text.

The product of the reaction catalyzed by 4,6-dehydratase HS41.20 was characterized by ESI-MS and ^1^H NMR spectroscopy. When CDP-Glc (**4**) was dehydrated in H_2_O, the product exhibited an *m*/*z* for the [M-H^+^]^−^ anion of 546.06, fully consistent with the formation of compound **5**. When the reaction was conducted in D_2_O, the [M-H^+^]^−^ anion exhibited an *m*/*z* of 547.06, consistent with the formation of product **5a**, which now contains a deuterium at C5 due to the reduction of the intermediate with the transiently formed NADH in D_2_O (Scheme S1). The ESI-MS experiments also indicated the appearance of additional peaks at *m*/*z* values of 564.07 and 565.07 when the reactions were conducted in H_2_O and D_2_O, respectively, consistent with the hydration of the newly formed keto group of products **5** and **5a** (Figure 5a,b). In each case, the ratio of the hydrate to the keto-form is approximately 4:1. The ^1^H NMR spectra of products **5** and **5a** are shown in Figures S7 and S8, respectively. The C6 methyl group resonates at 1.21 ppm as a singlet in compound **5a** due to the presence of deuterium at C5. The corresponding methyl group of the hydrate resonates slightly downfield at 1.17 ppm. The intensity ratio of the two resonances is ∼1:5 in favor of the hydrate. A similar ratio has been observed previously for the formation of UDP-4-keto-d-glucose.^17^

**Figure 5:**
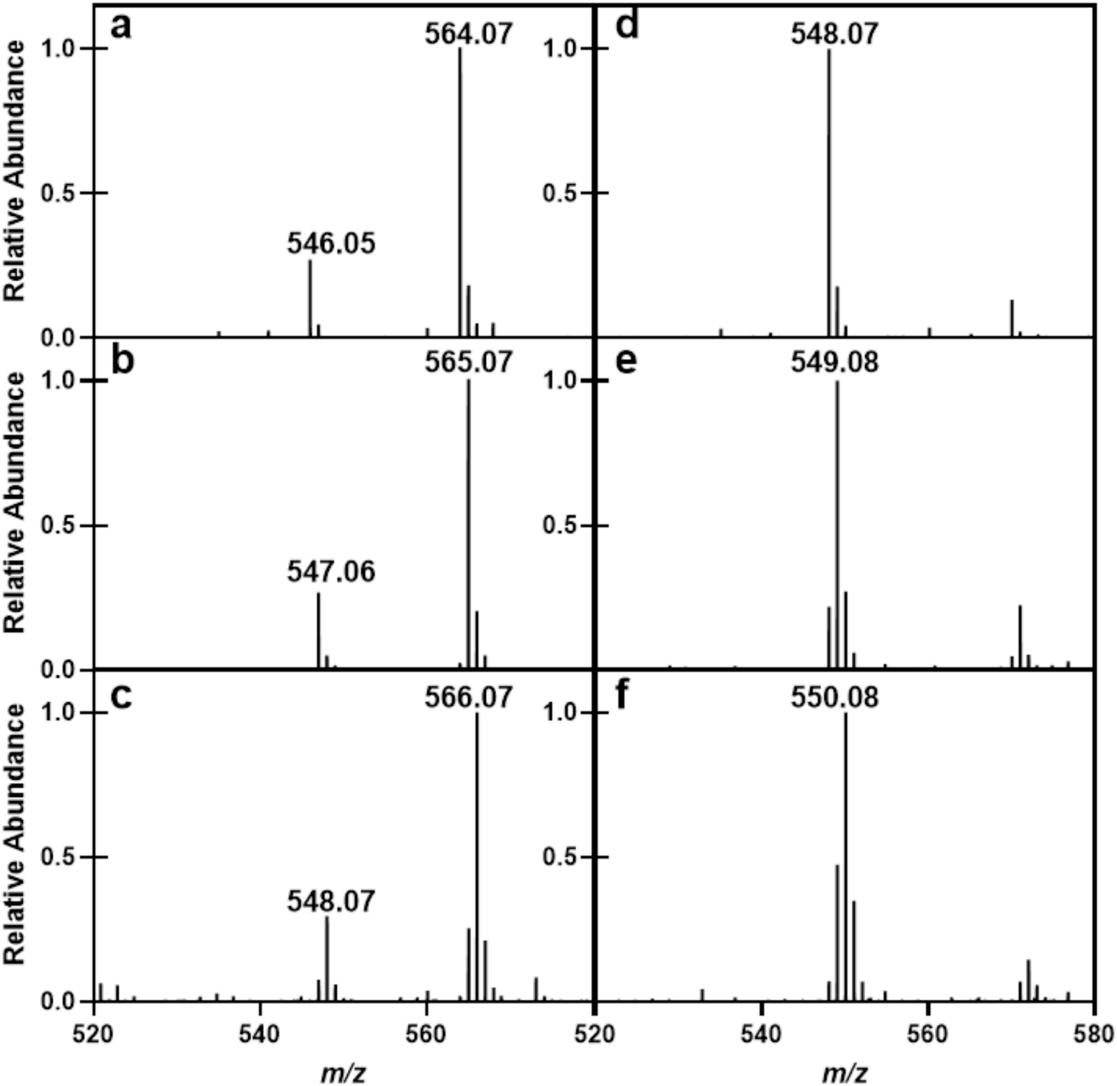
ESI-MS spectra of CDP-4-keto-6-deoxy-glucose (**5**, **5a**, and **5b** and the corresponding hydrates) and CDP-d-fucose/CDP-6-deoxy-l-altrose (**7**, **7a**, and **7b**). (a) CDP-4-keto-6-deoxy-glucose (**5**); (b) CDP-4-keto-6-deoxy-d-glucose made in D_2_O (**5a**); (c) CDP-4-keto-6-deoxy-d-glucose made in D_2_O and in the presence of the 3,5-epimerase (**5b**); (d) CDP-d-fucose (**7**); (e) CDP-d-fucose made in D_2_O (**7a**); (f) Mixture of CDP-d-fucose (**7b**) and CDP-6-deoxy-l-altrose (**8b**) made in D_2_O.

The values of *k*_cat_ and *k*_cat_/*K*_m_ for the 4,6-dehydratase were determined by measuring the rate of formation of compound **5** in a coupled assay with an excess of the C4-reductase in the presence of NADPH. At pH 7.5, *k*_cat_ and *k*_cat_/*K*_m_ were determined to be 0.47 ± 0.01 s^–1^ and 2.2 (±0.2) × 10^5^ M^–1^ s^–1^, respectively. The double reciprocal plot is presented in Figure S9. These experiments collectively demonstrate that HS41.20 catalyzes the NAD^+^-dependent 4,6-dehydration of CDP-Glc (**4**) to CDP-4-keto-glucose (**5**).

### CDP-4-Keto-6-deoxy-d-glucose-3,5-epimerase

The epimerization of CDP-4-keto-6-deoxy-glucose (**5**) is catalyzed by the third enzyme in the pathway. After the epimerase is added to compound **5** in D_2_O, ESI-mass spectrometric analysis of the product(s) indicated the incorporation of two deuterons with an *m*/*z* value of 548.07 for the [M-H^+^]^−^ anion (Figure 5c). This result demonstrates that the enzyme catalyzes the exchange of protons with solvent deuterons at both C3 and C5 and forms a mixture of compounds **5b** and **6b** (Scheme S1). The ^1^H NMR spectra of the reaction mixtures after the incubation of compound **5** with the 3,5-epimerase in H_2_O and D_2_O are presented in Figures S10 and S11, respectively. In both spectra, it appears that the major compound is the starting material CDP-4-keto-6-deoxy-glucose (**5**). This is exemplified by the major resonances for the H6-methyl group at ∼1.11 ppm and the H1-anomeric hydrogen at ∼5.39 ppm. There are, however, two additional doublets that appear between 1.32 and 1.41 ppm in Figure S10 (and singlets in Figure S11) that may arise from the methyl group in either of the C3 or C5 epimerized products.

The rate constant for the isomerization of C3 and C5 in compound **5a** was estimated by measuring the exchange rate for the loss of deuterium at C5 when the reaction was conducted in H_2_O and incorporation of deuterium at C3 when the reaction was conducted in D_2_O (Scheme 1). In each case, the 3,5-epimerase was incubated with compound **5a**, and at various time points, aliquots were removed and the relative concentrations of the undeuterated, monodeuterated, and dideuterated products were determined by ESI-MS (Figure S12). At an initial concentration of 1.0 mM **5a** using 1.5 μM 3,5-epimerase in H_2_O, the calculated rate constant for the equilibration of **5a** to the undeuterated product was determined to be 1.3 ± 0.2 s^–1^. Conversely, at an initial concentration of 0.5 mM compound **5a** using the same amount of enzyme in 90% D_2_O, the observed rate constant for the equilibration of **5a** with the dideuterated product was determined to be 0.31 ± 0.06 s^–1^. These studies demonstrate that HS41.19 is the 3,5-epimerase that enables the conversion of compound **5** to **6**.

**Scheme 1:**
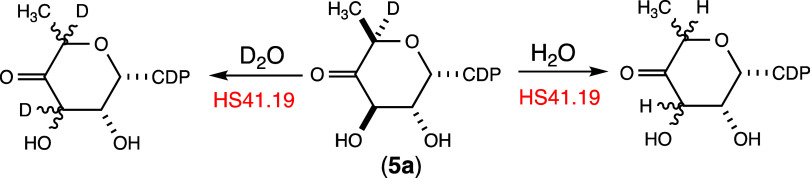
Solvent Exchange Reactions Catalyzed by the 3,5-Epimerase (HS41.19)

### CDP-4-Keto-6-deoxy-reductase

Reduction of the two 4-keto-6-deoxy-CDP sugars (compounds **5** and **6**) is catalyzed by apparent C4-reductase (HS41.18) using NADPH as the cofactor. When we incubated compound **5** with the enzyme and NADPH, the product was found by ESI-MS to have an *m*/*z* for the [M-H]^−^ anion of 548.07 (Figure 5d), fully consistent with the formation of CDP-d-fucose (**7**). When the C5-deuterated **5a** is used as the substrate, the value of *m*/*z* for the [M-H]^−^ anion was determined to be 549.07, again consistent with the formation of the deuterated product **7a** (Scheme S1). If the C4-reductase is added to a mixture of **5b** and **6b** (formed from **5** in the presence of the 3,5-epimerase in D_2_O), the *m*/*z* for the presumptive product mixture was found to be 550.08, consistent with the formation of products **7b** and **8b**.

The structure of CDP-d-fucose (**7**) was confirmed by ^1^H NMR, as illustrated for the isolated compound in Figure S13, where the resonances for the hydrogens at H1–H4 are clearly separated from one another. When compound **5a** was used as the substrate, the resonance for H5 is lost, in addition to the proton coupling to the methyl group (Figure S14). When compound **5** is reduced by the C4-reductase in the presence of the 3,5-epimerase, two products are formed that can be separated via chromatography using the Carbopac PA1 column (Figure 6a,b). The first compound has a retention time identical to that of the CDP-d-fucose (**7**) formed in the absence of the epimerase, while the more abundant compound with a longer retention time is the C5-epimer (CDP-6-deoxy-l-altrose, **8**). This compound was isolated, and the ^1^H NMR spectrum is presented in Figure S15. If the same set of experiments were conducted in D_2_O, the isolated product (**8b**) had lost resonances for both H3 and H5 (Figure S16). These results show that C4-reductase reduces the equilibrium mixture of compounds **5** and **6** regardless of stereochemistry at C5, while apparently being selective at C3. It was also found that the ratio of CDP-d-fucose (**7**) and CDP-6-deoxy-l-altrose (**8**) could be altered, depending on the concentration of the 3,5-epimerase. When the reductase and epimerase were at an equal concentration, 96% of the final product was CDP-6-deoxy-l-altrose (Figure 6b) but approximately equal in concentration when the 3,5-epimerase and C4-reductase were in a ratio of 1:27 (Figure 6c).

**Figure 6:**
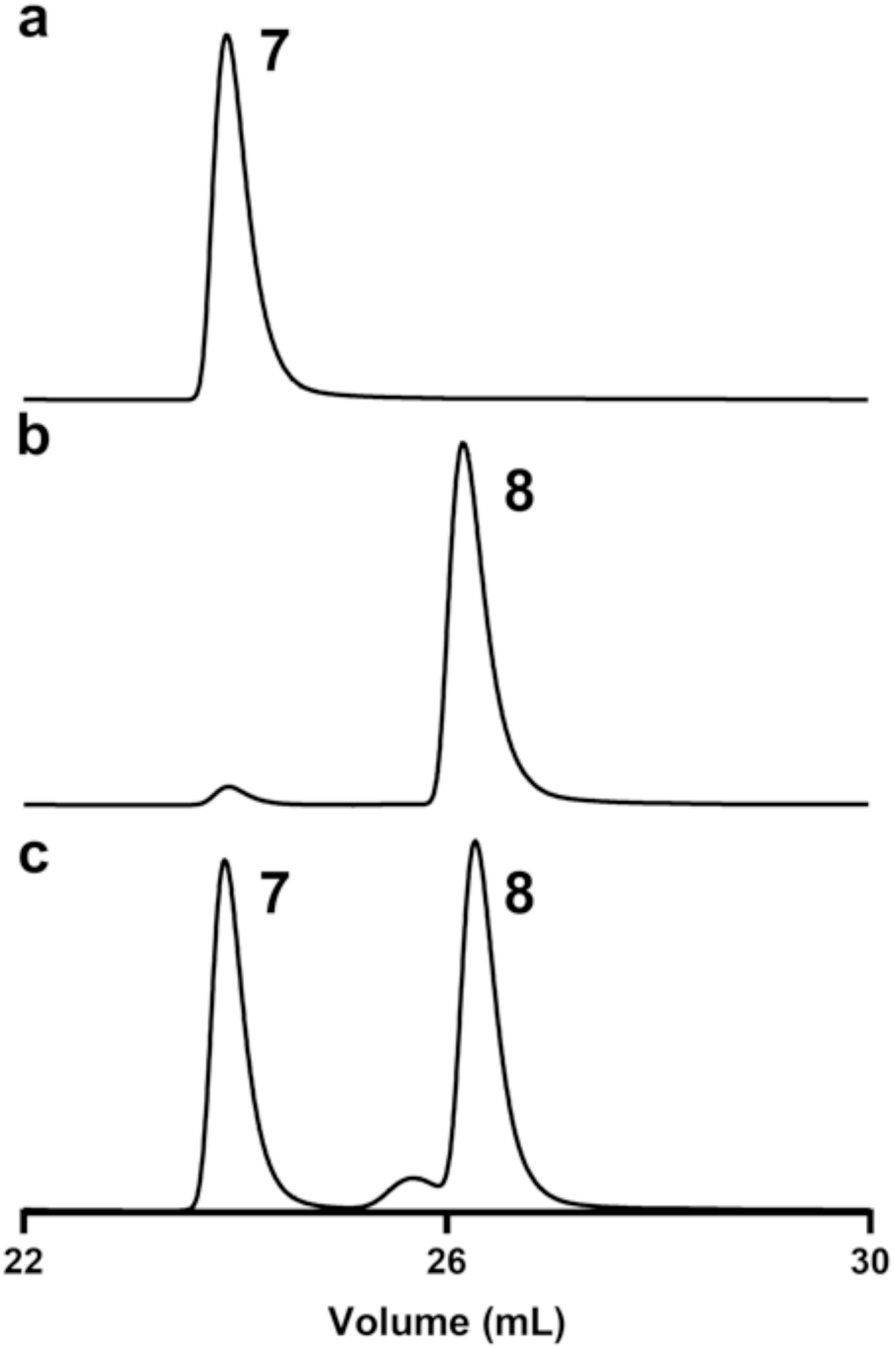
Chromatographic separation of reaction products using a Carbopac PA1 column. (a) CDP-d-fucopyranose (**7**); (b) mixture of CDP-d-fucopyranose (**7**) and CDP-6-deoxy-l-altropyranose (**8**) formed from the reduction of compound **5** with NADPH in the presence of equal concentrations of the 3,5-epimerase and the C4-reductase; (c) mixture of CDP-d-fucopyranose (**7**) and CDP-6-deoxy-l-altropyranose (**8**) formed from the reduction of compound **5** with NADPH in the presence of the 3,5-epimerase and the C4-reductase in the ratio of 1:27. Additional details are found in the text.

The values of *k*_cat_ and *k*_cat_/*K*_m_ for the C4-reductase were first determined by the addition of enzyme to variable concentrations of compound **5** in the presence of NADPH for the synthesis of CDP-d-Fuc (**7**). Under these conditions, the values of *k*_cat_ and *k*_cat_/*K*_m_ were determined to be 0.22 ± 0.01 s^–1^ and 1.5 (±0.1) × 10^4^ M^–1^ s^–1^, respectively (Figure S17). In the presence of the 3,5-epimerase, where the C4-reductase can reduce the equilibrium mixture of compounds **5** and **6**, the values of *k*_cat_ and *k*_cat_/*K*_m_ were determined to be 0.17 ± 0.01 s^–1^ and 1.2 (±0.1) × 10^4^ M^–1^ s^–1^, respectively (data not shown).

### CDP-Pyranose/Furanose Mutase (HS41.17)

The last step in the proposed biosynthetic pathway is the enzymatic conversion of CDP-d-fucose (**7**) and CDP-l-6-deoxy-altrose (**8**) to the corresponding furanoses **9** and **10** by the mutase HS41.17. The isolated enzyme was bright yellow in color, indicating the tight binding of the required flavin cofactor.^18,19^ The cofactor was removed from the as-purified enzyme by heat denaturation, and the supernatant solution exhibited the characteristic absorbance maxima at 260 and 450 nm for FAD, as illustrated in Figure S18. The cofactor was subsequently demonstrated to be FAD by anion exchange chromatography at pH 6.0 (data not shown). Since the equilibrium constant for the interconversion of pyranose **7** and furanose **9** favors **7**, the catalytic activity of the mutase was probed by incubation of chemically synthesized **9** with HS41.17 in the presence of dithionite to reduce the tightly bound FAD. The products were quantified after separation via chromatography using the Carbopac PA1 column, and the elution profiles for the reactions are presented in Figure 7. The control (Figure 7a) shows the elution profile for compound **9**, and Figure 7b shows the formation of the corresponding pyranose **7**. The ratio of **7** and **9** at equilibrium is 92:8. A nearly identical set of experiments was conducted to demonstrate the conversion of chemically synthesized CDP-6-deoxy-l-altrofuranose (**10**) to the corresponding pyranose (**8**). The elution profiles are shown in Figure 7c,7d, respectively. At equilibrium, the ratio of **8** and **10** is ∼50:50. When the reaction was initiated with CDP-6-deoxy-l-altropyranose (**8**), as illustrated in Figure 7e, the same ratio of substrate (**8**) and product (**10**) was observed as when the reaction was initiated with **10** (compare Figure 7d,7f). The enzyme-catalyzed rate for the conversion of furanose **9** to pyranose **7** was determined by monitoring the formation of **7** after the separation of substrate and product via chromatography. At a fixed concentration of furanose **9** of 1.0 mM and an enzyme concentration of 5.0 nM, the apparent rate constant was determined to be 9 ± 1 s^–1^.

**Figure 7:**
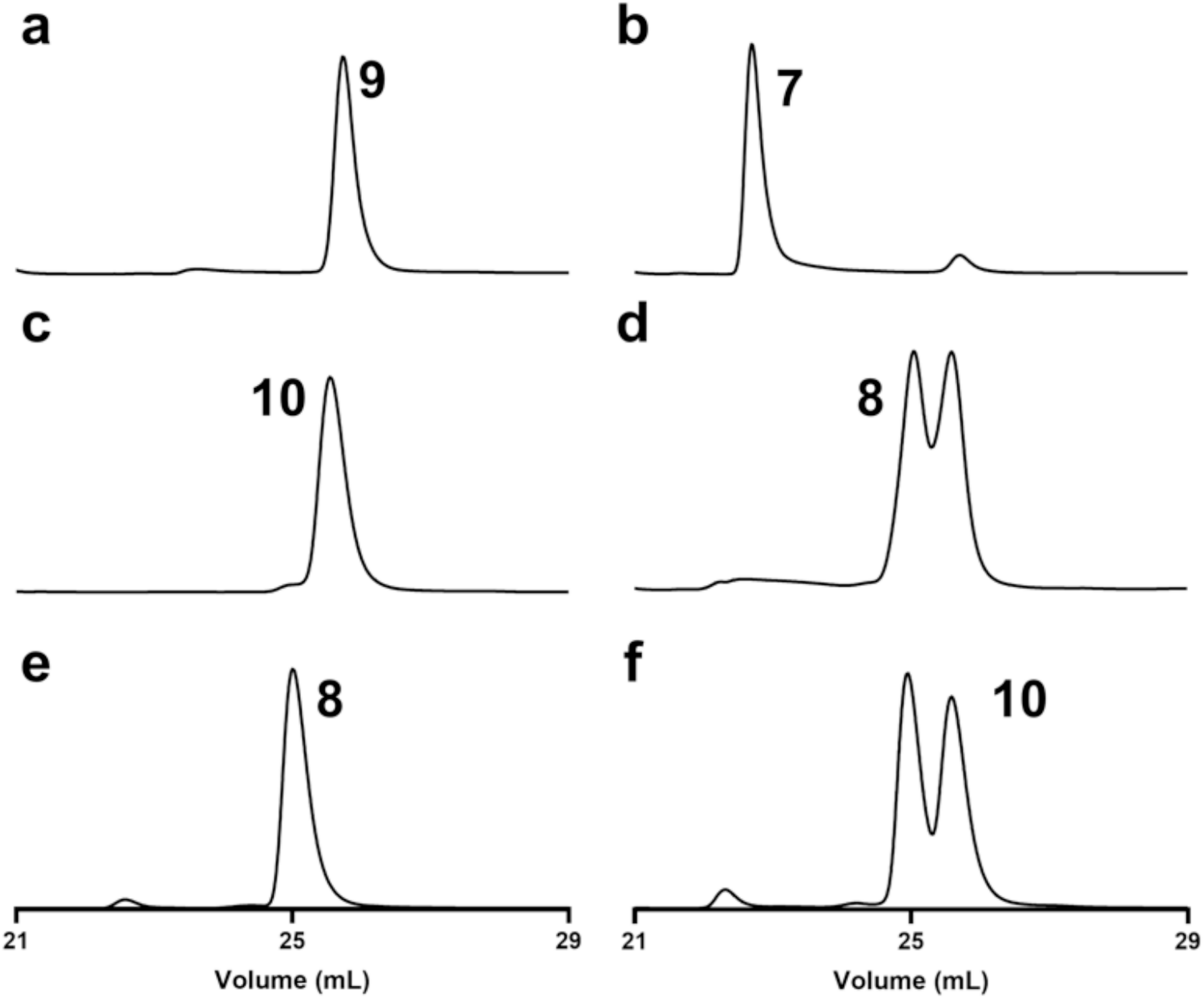
Chromatographic separation of compounds **7–10** using a Chromopac PA1 column to monitor the catalytic activity of pyranose/furanose mutase HS41.17. (a) chemically synthesized CDP-d-fucofuranose (**9**); (b) mixture of products (**7** and **9**) after the incubation of HS41.17 with CDP-d-fucofuranose (**9**); (c) chemically synthesized CDP-6-deoxy-l-altrofuranose (**10**); (d) mixture of products (**8** and **10**) after the incubation of HS41.17 with CDP-6-deoxy-l-altrofuranose (**10**), (e) enzymatically synthesized CDP-6-deoxy-l-altropyranose (**8**); and (f) mixture of products **8** and **10** after incubation of HS41.17 with CDP-l-6-deoxy-altropyranose (**8**) with CDP-d-fucofuranose (**9**). Additional details are found in the text.

### Positional Isotope Exchange Catalyzed by the Mutase HS41.17

It has previously been demonstrated that the pyranose/furanose mutases require the enzyme-bound flavin to be reduced for catalytic activity.^18–21^ The generally accepted reaction mechanism postulates that N5 of FADH_2_ attacks C1 of the XDP-pyranose to form a covalent adduct between the flavin and the substrate, coupled with expulsion of XDP.^18,19,21^ Subsequent iminium ion formation results in the liberation of the C5-hydroxyl group and subsequent reattack by the C4-hydroxyl group to form the corresponding furanose intermediate with the reduced flavin. In the last step, the tightly bound XDP then attacks the flavin-furanose intermediate to form the XDP-furanose. This mechanism is summarized in Figure S19.

The reaction mechanism for the pyranose/furanose mutase (HS41.17) was further interrogated using positional isotope exchange (PIX), as illustrated in Figure 8.^13,18,20^ In this experiment, the ^18^O-labeled CDP-α-d-fucopyranose (**7x**) was incubated with the dithionite-reduced enzyme to form the transient substrate-flavin adduct and ^18^O-labeled CDP. Rotation about the β-phosphoryl group will torsionally scramble the ^18^O-label, and reformation of the CDP-d-fucopyranose will positionally exchange the ^18^O-label from the C1-β-phosphoryl bridge position to the two possible β-nonbridge positions (**7y** and **7z**). The ^31^P NMR spectrum for the β-phosphoryl group of the ^18^O-labeled CDP-d-fucopyranose (**7x**) is presented in Figure 9a, and the equilibrium reaction mixture after the addition of the mutase is shown in Figure 9b. The latter spectrum clearly shows the formation of the isotope scrambled products **7y** and **7z** by the 0.016 ppm upfield chemical shift for approximately 2/3 of the total ^31^P NMR signal for the β-phosphoryl group.^15,22^

**Figure 8:**
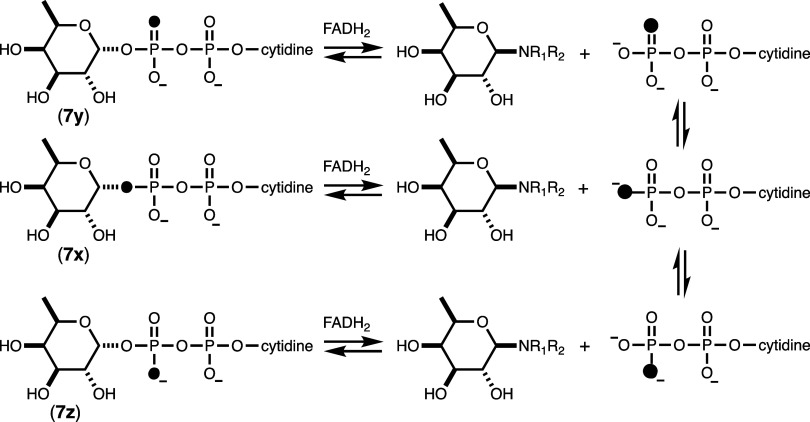
Positional isotope exchange catalyzed by CDP-d-fucopyranose mutase (HS:41.17).

**Figure 9:**
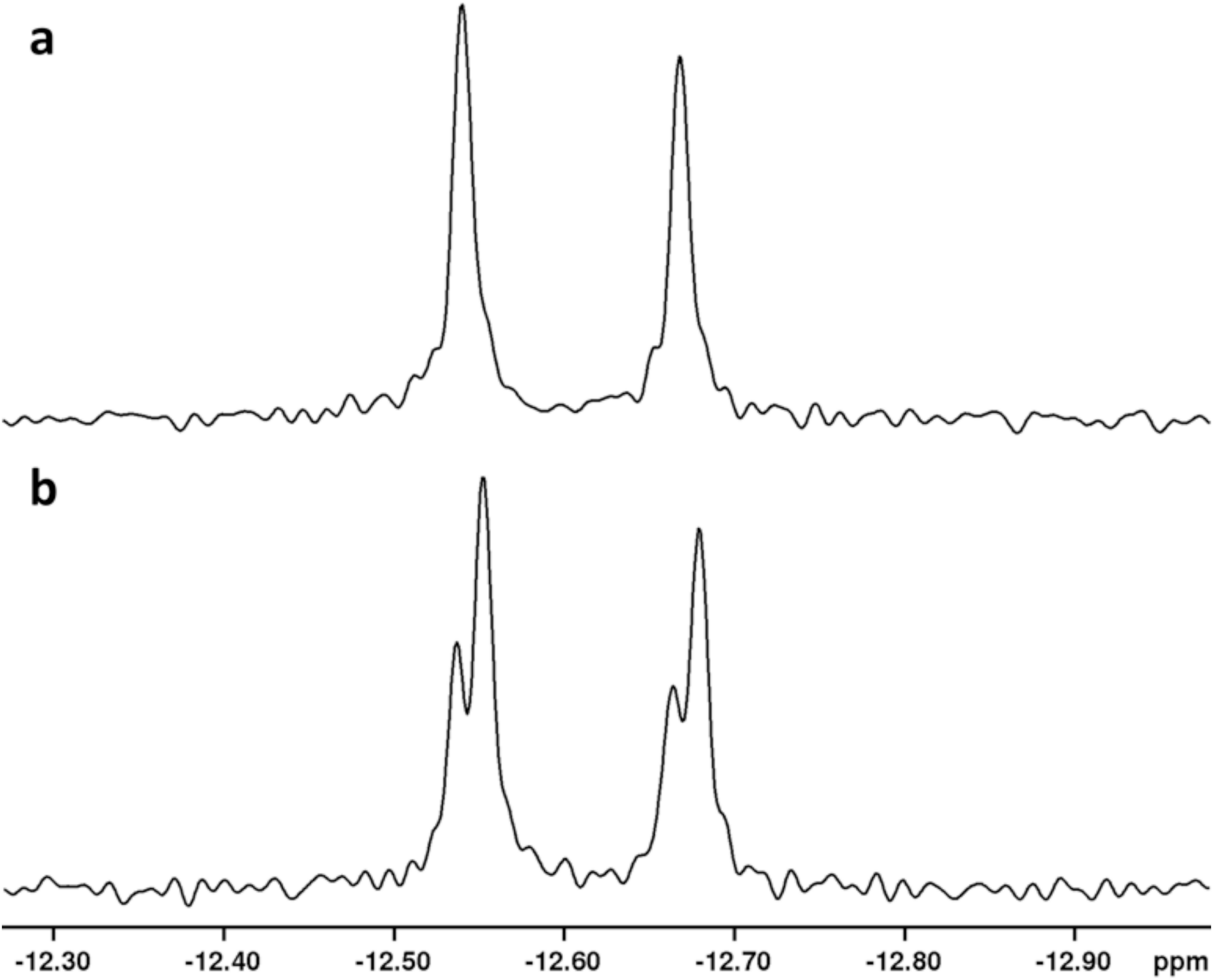
^31^P NMR analysis of the PIX reaction catalyzed by CDP-pyranose/furanose mutase (HS41.17). (a) ^31^P NMR spectrum of ^18^O-labeled CDP-d-fucopyranose (**7x**) showing the β-phosphoryl group; (b) corresponding ^31^P NMR spectrum for the β-phosphoryl group of CDP-d-fucopyranose after the addition of HS41.17 and showing the formation of products **7y** and **7z**. Additional details are provided in the text.

### Biosynthesis of CDP-d-fucose and CDP-6-deoxy-l-altrose

We have now demonstrated that the HS:41 serotype of synthesizes CDP-d-fucose and CDP-6-deoxy-l-altrose by a 5-step biosynthetic pathway starting with CTP and Glc1P as initially proposed in Figure 3. The pathway is initiated by the formation of CDP-Glc (**4**) from CTP and Glc1P (**3**). The CDP-Glc (**4**) is then dehydrated to form CDP-4-keto-6-deoxy-d-Glc (**5**). In the absence of the 3,5-epimerase (HS41.19), a C4-reductase (HS41.18) then catalyzes the formation of CDP-d-fucose (7), and the mutase (HS41.17) catalyzes the pyranose to furanose ring contraction to generate CDP-d-fucofuranose (**9**). In the presence of the 3,5-epimerase, the C4-reductase predominantly catalyzes the formation of CDP-6-deoxy-l-altrose (**8**), and the same mutase now catalyzes the formation of CDP-6-deoxy-l-altrofuranose (**10**). This pathway is interesting since two of the enzymes (the C4-reductase and pyranose/furanose mutase) are promiscuous in the sense that the absolute stereochemistry at C5 is relatively unimportant. Equally interesting is the fact that the 3,5-epimerase controls the final reaction product and that a poly-G/C tract is found within the gene for HS41.19.^23^ It is likely but unproven that the poly-G/C tract controls the ultimate expression levels of the 3,5-epimerase and thus the final sequence of the capsular polysaccharide.

A poly-G tract also appears in the gene cluster for the biosynthesis of GDP-6-deoxy-d-*altro*-heptose that may control whether the gene for HS41.13 or HS41.12 is expressed.^11,23^ HS41.13 is a C4-reductase that catalyzes the NADPH-dependent reduction of GDP-6-deoxy-4-keto-d-*arabino*-heptose to GDP-6-deoxy-d-*altro*-heptose, whereas HS41.12 is a C4-reductase/3,5-epimerase that converts GDP-6-deoxy-4-keto-d-*lyxo*-heptose to GDP-6-deoxy-l-*galacto*-heptose.^9^ Therefore, these poly-G/C tracts may enable to rapidly alter the sequence of the capsular polysaccharide to avoid the host immune response.

## Conclusions

We identified and functionally characterized the five enzymes responsible for the biosynthesis of CDP-d-fucofuranose and CDP-6-deoxy-l-altrofuranose that are utilized in the construction of the CPS in the HS:41 serotype of (Figure 3). The first enzyme uses CTP and d-glucose-1-phosphate to generate CDP-d-glucose (**4**). The second enzyme is an NAD^+^-dependent 4,6-dehydratase that converts CDP-d-glucose (**4**) to CDP-4-keto-6-deoxy-d-glucose (**5**). A C4-reductase can then reduce the carbonyl group at C4 of product **5** to generate CDP-d-fucopyranose (**7**), which is then converted to CDP-d-fucofuranose (**9**). Alternatively, a 3,5-epimerase catalyzes the C5-isomerization of CDP-4-keto-6-deoxy-d-glucose (**5**), and the C4-reductase catalyzes the NADPH-dependent reduction of this product to CDP-6-deoxy-l-altropyranose (**8**). This product reacts with the pyranose/furanose mutase to then form CDP-6-deoxy-l-altrofuranose (**10**). In the chemically determined structure of the CPS from the HS:41 serotype of , it has been reported that the ratio of d-fucose to 6-deoxy-l-altrose is approximately 1:3, and this ratio now appears to be dictated by the presence of the 3,5-epimerase. This is the first pathway for the biosynthesis of 6-deoxy-l-altrose to be determined from any organism.

## Supplementary Material



## Notes

Amino acid sequences of the purified proteins and ESI-MS and NMR spectra of enzyme-catalyzed reaction products (PDF)
