# Supplementary material for: Biosynthesis of CDP-α‑d‑fucofuranose and CDP-β‑l‑6-deoxy-altrofuranose for the Capsular Polysaccharides of
Source: Biochemistry. 2025 Aug 8;64(16):3623–33. doi: 10.1021/acs.biochem.5c00296 (PMC12369002; doi:10.1021/acs.biochem.5c00296)
Supplement: Supplementary file 1 [file bi5c00296_si_001.pdf]

Supplementary Information

Biosynthesis of CDP-α-D-Fucofuranose and CDP-β-L-6-Deoxy-Altrofuranose for the Capsular Polysaccharides of *Campylobacter jejuni*

Max Errickson Simons[ψ], Tamari Narindoshvili[φ] and Frank M. Raushel[ψ, φ, *]

[ψ]Department of Biochemistry & Biophysics, Texas A&M University, College Station, TX 77843, USA

[φ]Department of Chemistry, Texas A&M University, College Station, TX 77843, USA

*Contact Information

e-mail: raushel@tamu.edu

phone 1-979-845-3373

HS:41.21 (Uniprot id: Q5M6T0)

MMKVLILAGGLGTRLSEETSLKPKPMVEIGGKPILWHIMKIYSYYGFNDFIILTGYKGHIIKDYFINYYTQYSDITV
DMSDNSVQIHNTRHEPWKVTMLYTGQDSMTGGRILYAKPYVGNETFMLTYGDGVSDVNIKELINFHKSHSKAITMTS
VLPEGKFGALDIDNDTNCIKNFTEKPKGDTNLNNTGWINGGFFVCEPKIFDYIKEGDETVFEQDPLRNLAQDSELYS
YKHYGFWKCMDTLKDKNDLTRMWINNKAPWALWLNRENLYFQGHLEHHHHHH

HS:41.20 (UniProt id: Q5M6T1)

MGSSHHHHHHSSENLYFQGHMLFKIYENTKVFLTGHTGFKGSWLGLWLHNLGAKVDGYSLQPNAKINHFDLLGGERL
YENSYFSDINDAIKLEKALINSDPEIIFHLAAQPLVRKSYQDPYNTFQTNTIGTLNILNISRKLKNLKAIVLITTDK
VYKNKEWFWGYRESDTLGGYDPYSASKACAELVIDSMRQSFFNANDFGSKHQILIASARAGNVIGGGDWSEDRLIPD
IIKACIGNKIVEIRNPKSTRPWQHVLEPLYGYLLLGTKLLQGHTEFATSFNFGPEVSGNLRVEEVLKIAKNFWDKIS
YQHKIDLNAPHEANLLMLDTSKAKKMLSWNSLLTAEESIKMTIEWYKSFYNKKELISVKQLKKIIEGL

HS:41.19 (UniProt id: Q5M6T2)

MGSSHHHHHHSSENLYFQGHMSRFNFMKTCIEGVYIIKPKPICDERGYFERYFCTNDFEEIGMKKPIIQINHSKTIG
KGSIRGMHYQIPPFCETKIVRCLKGSILDVAIDIRKNSPTFLQYFSIELNEVNNKYLYIPEGFAHGFQVLSDEAEIL
YLVTQEFNSSADRGINPFDKAIISTNNFYQRNRHNVFENYKKKGGGIKWPLGSGNISRKDLDRQYIDNNFIGIEI

HS:41.18 (UniProt id: Q5M6T3)

MGSSHHHHHHSSENLYFQGHMKILITGGTGFIGSNFIRKYHNKYDIIALVRKNSDISNIKDKCVVYTYDKDIDKLLL
FLQKENIQGVVHLAALYFSKHSPSDVKKLFNSNILFGLELLDIFVQLKIGFFINTVTFSQFANSIHYKPKTLYDATK
QAFMDLTNFYSSYFDIVNLMIYDNYGNNDIRPKIFNFWKKISLNEEILDMSPGEQKIDITHIDDVIDGFDILISMCI
EKKIINNQIYTLENTRYTLKELANLFEQYTKRKINIKWGAREYSPNEIMEPISSKSSSKLMRLPNWKPKISLQEGLN
NFLKEL

HS:41.17 (UniProt id: Q5M6T4)

MGSSHHHHHHSSENLYFQGHMDILVIGSGITGATIARILAEKGNNVTIWERRNHIGGNMYDYVDEYGILVHKYGPHT
FHTSKRYLYDFVLKYSEWIDYKLVCGAKIDDKIVSVPFNFHTIDTFYNKDESKFLKQKIKEEFGERKKITVFEALNS
KCDLIKVYANFLFEKDYKPYTSKQWGIPLDKIDVSIFQRVPLNLSYENGYFDDKFQIMPKISYSYFFKNLLQHKNIK
VVLNKDALDSLRIIGNNINLYGNKITIPVIYTGALDELFGYKYGKLPYRTLEFEWRHENIESKQPFPVVAYPFECYT
RITEYKKLPHQKAQGTSYALEYSLEYNKEKGHEPYYPVMTQDSSYIYEKYKQESRFVKNLYPVGRLADFKYYNMDQA
LDRALEIANIL

Figure S1: Amino acid sequences of the enzymes produced for this investigation. The polyhistidine purification tags are highlighted in yellow.

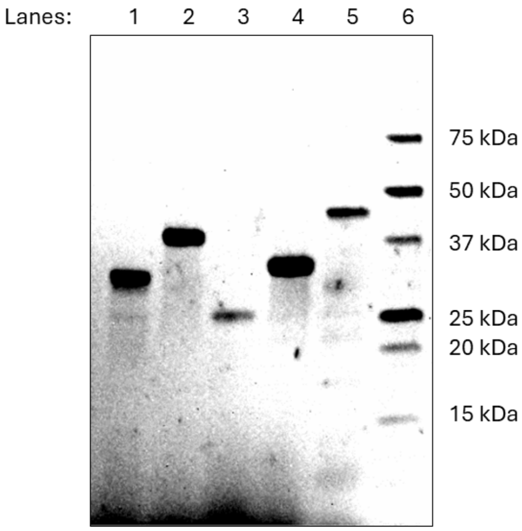

Figure S2: SDS-PAGE of the enzymes purified for this investigation. Lane 1: (HS41.21) α-D-glucose-1-phosphate cytidylyltransferase; Lane 2: (HS41.20) CDP-α-D-glucose 4,6-dehydratase; Lane 3: (HS41.19) CDP-α-D-4-keto-6-deoxy-glucose 3,5 epimerase; Lane 4: (HS41.18) CDP-hexose reductase; Lane 5: (HS:41.17) CDP-α-D-fucopyranose mutase; Lane 6: SDS protein ladder (Bio-Rad). The analysis was conducted using a Stain-Free SDS-PAGE gel from Bio-Rad which causes proteins to fluoresce when activated by UV light using a Gel Doc EZ Imager.

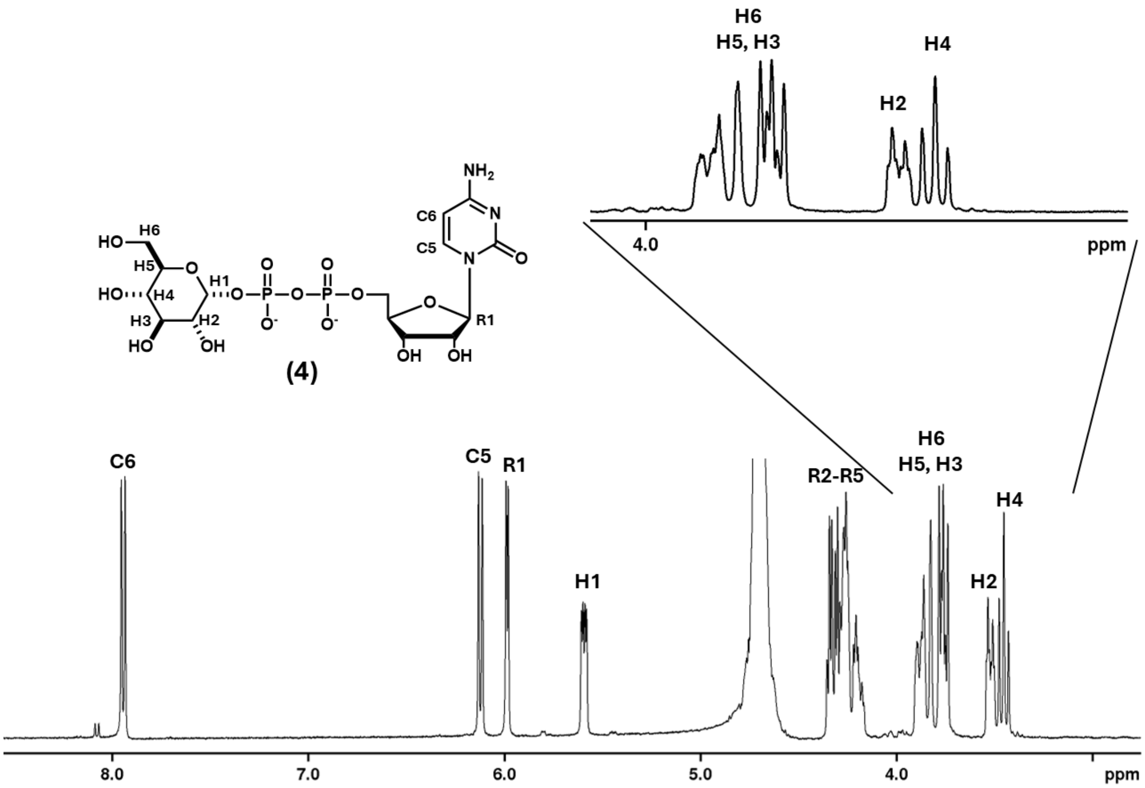

Figure S3: ^{1}H NMR spectrum of the enzymatically prepared CDP-Glc (**4**). The resonances assigned with an "R" refer to the protons from the ribose moiety while resonances assigned with an "H" refer to the glucose moiety, and resonances assigned with a C refer to the cytidine nucleotide base.

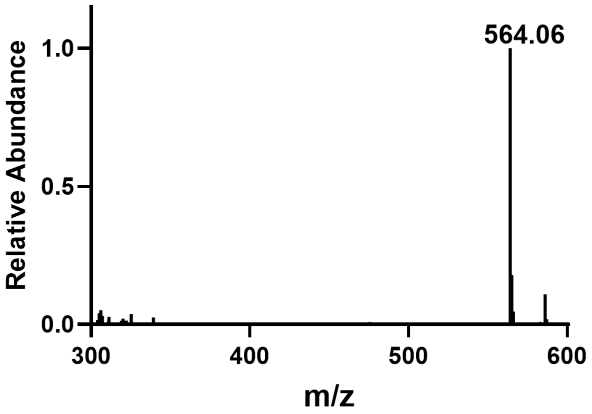

Figure S4: Negative ion ESI-MS of [M-H$^+$]$^-$ anion of CDP-α-D-glucose (**4**).

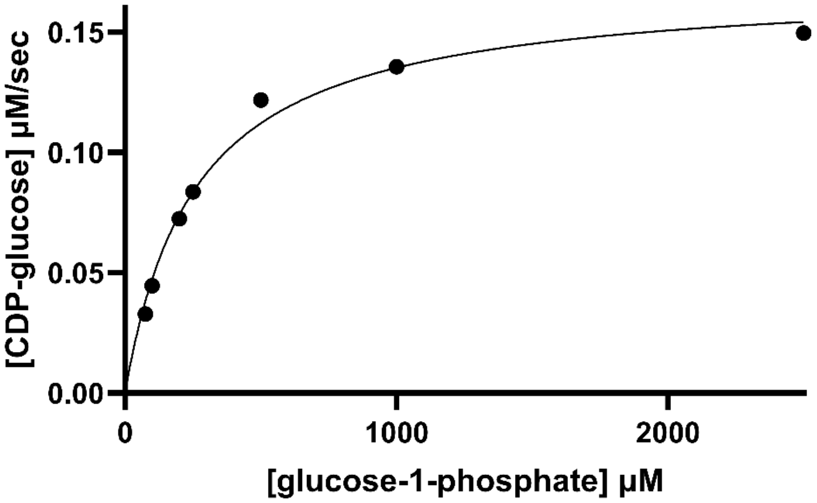

Figure S5: Michaelis-Menten plot for the reaction catalyzed by the D-glucose-1-phosphate cytidylyltransferase (HS41.21). All reactions contained 2.0 mM CTP, 3.0 mM $MgCl_2$, 0.36 µM Glc1P cydidylyltransferase, 60 nM inorganic pyrophosphatase and HEPES/K^+ (pH 7.5). Additional details are provided in the text.

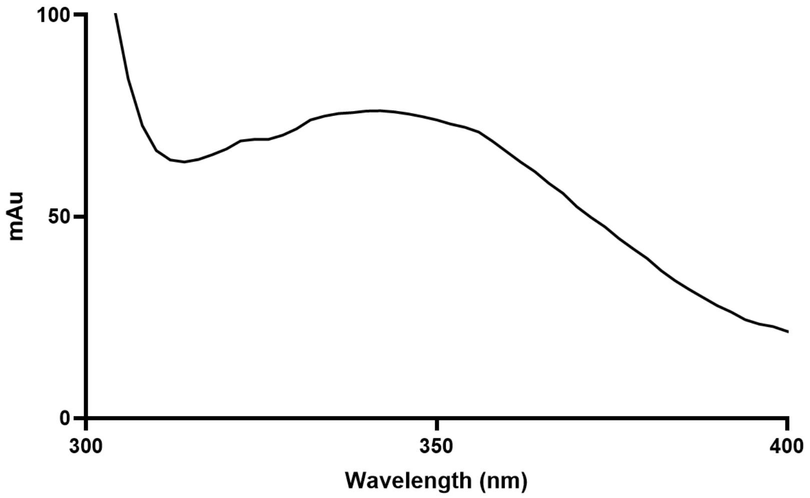

Figure S6: UV-vis spectrum of the as-purified CDP-α-D-glucose-4,6-dehydratase (HS:41.20) showing the presence of bound NADH at 342 nn. The concentration of enzyme was 18 μM.

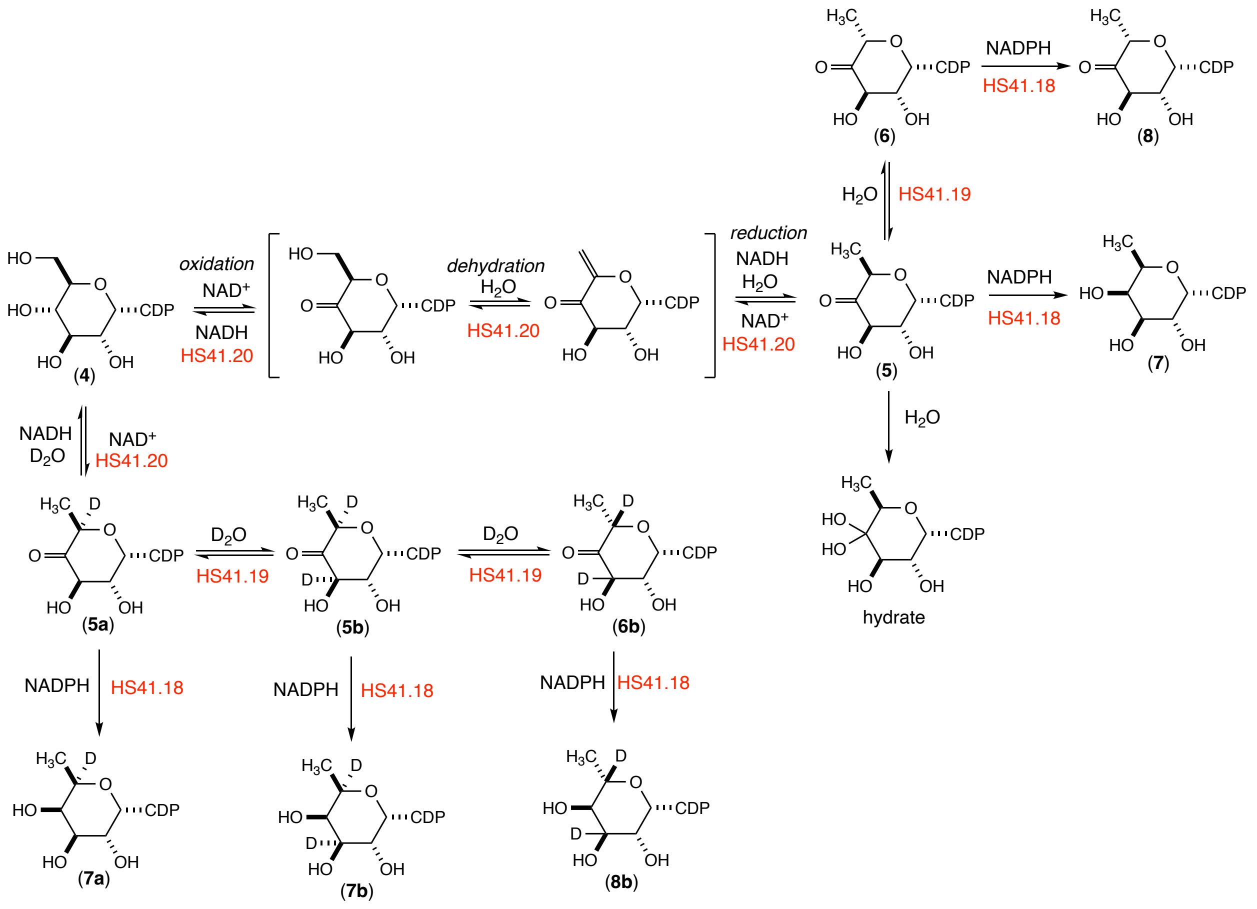

Scheme S1: Reaction scheme for the substrate and products made in this investigation.

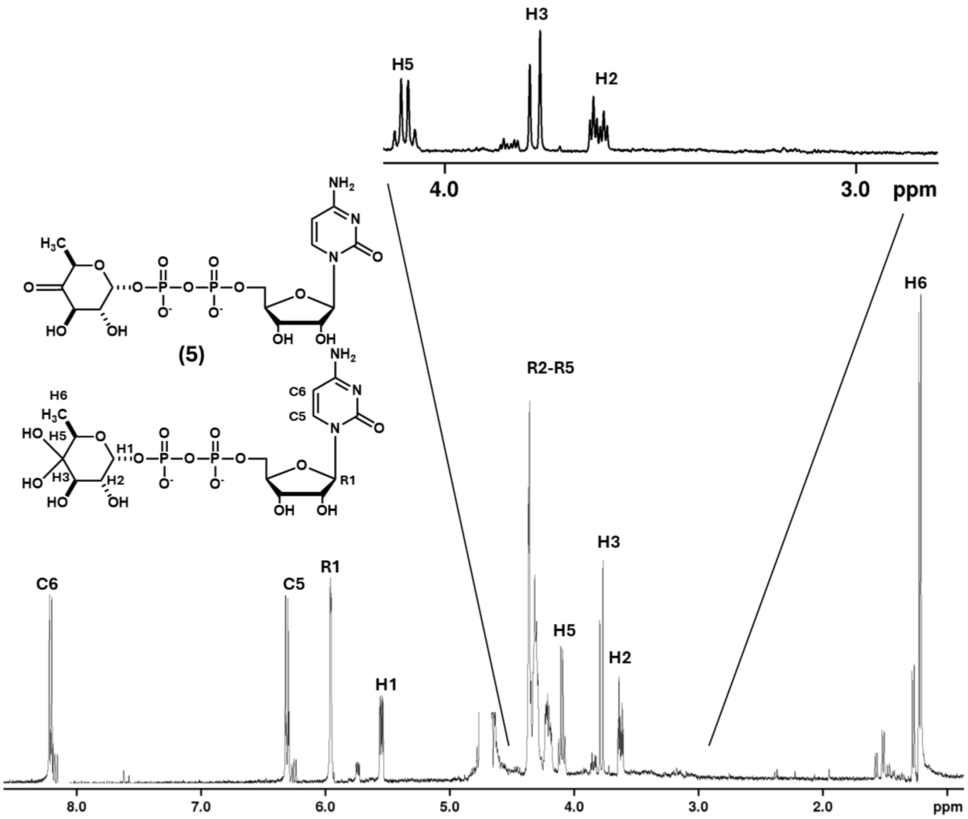

Figure S7: ^{1}H NMR spectrum of CDP-Glc dehydrated in H_2O to CDP-4-keto-6-deoxy-glucose (**5**). Peaks assigned with an R refer to the riboside protons, peaks assigned with an H refer to the hydrated 4-keto-fucose product and peaks assigned with a C refer to the cytidine nucleotide base.

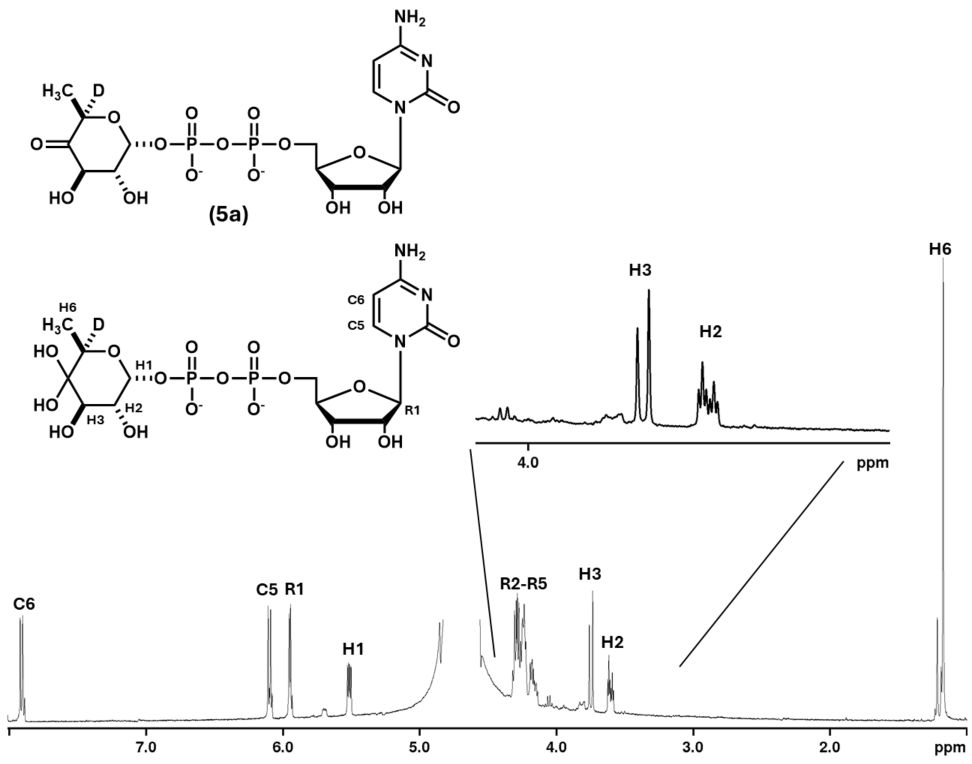

Figure S8: ^{1}H NMR spectrum of CDP-Glc dehydrated in D_2O to CDP-4-keto-6-deoxy-glucose (**5a**). The hydrogen at C5 has exchanged with deuterium from the solvent. Peaks assigned with an R refer to the riboside protons, peaks assigned with an H refer to the hydrated 4-keto-fucose product and peaks assigned with a C refer to the cytidine nucleotide base.

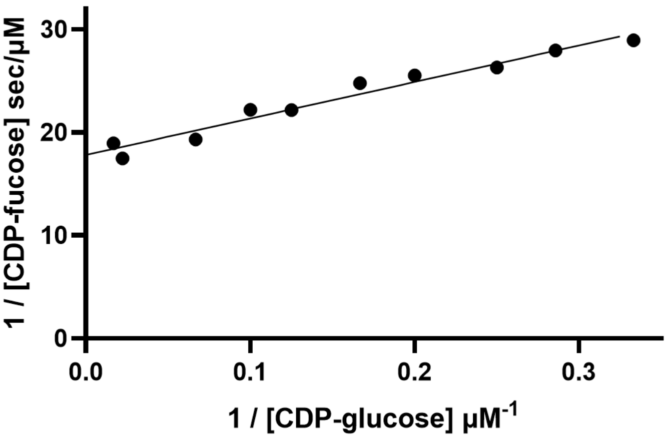

Figure S9: Double reciprocal plot for the reaction catalyzed by the CDP-Glc 4,6-dehydratase (HS:41.20). All reactions were conducted in 1-mL quartz cuvettes by monitoring the change in absorbance at 340 nm. All reactions contained 1.25 μM of the C4-reductase (HS:41.18), 0.16 mM NADPH, 120 nM CDP-Glc 4,6-dehydratase, 50 mM HEPES/K^+ (pH 7.5).

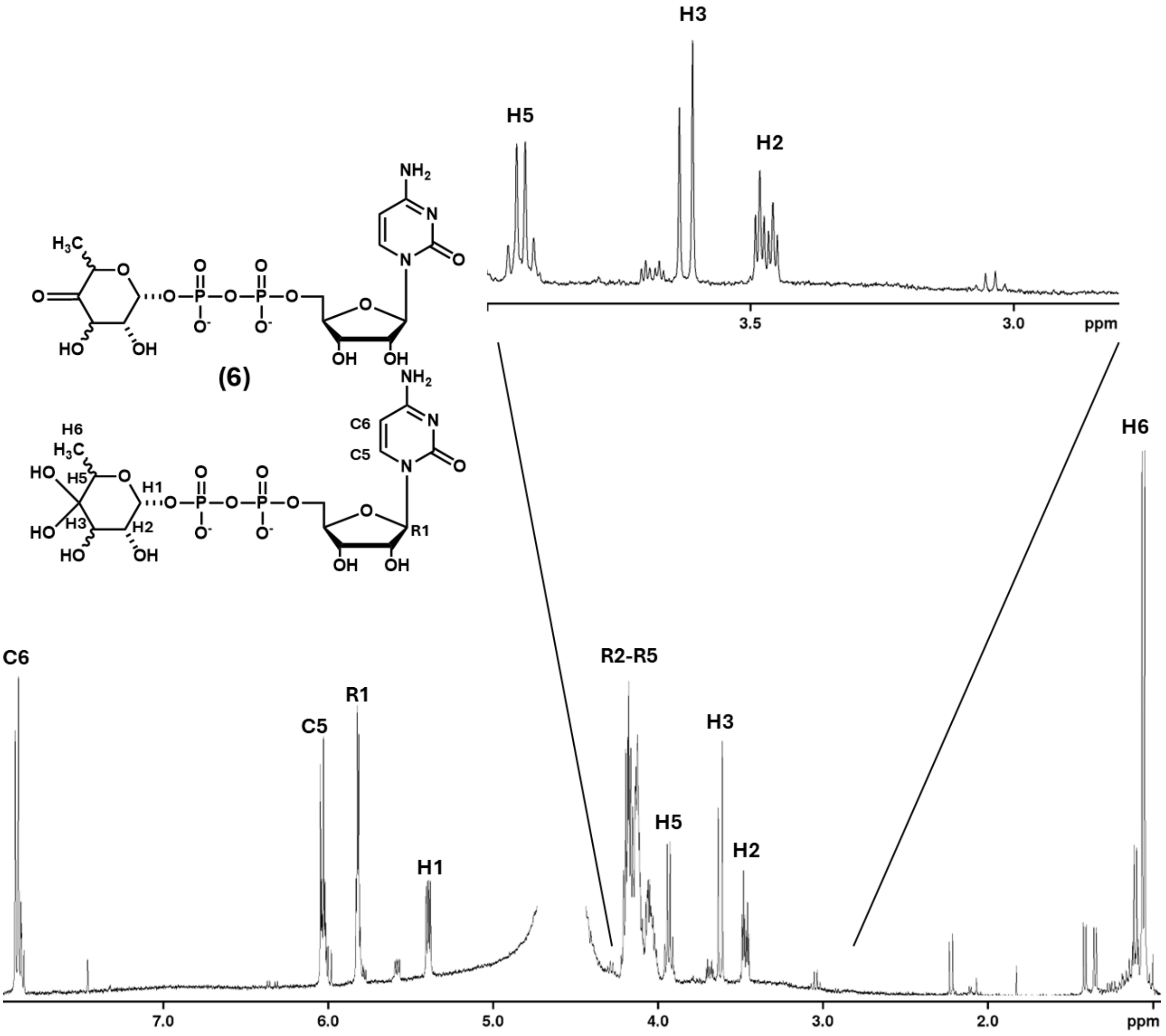

Figure S10: ^{1}H-NMR spectrum of compound **6** after incubation of compound **5** with the 3,5-epimerase in H_2O.

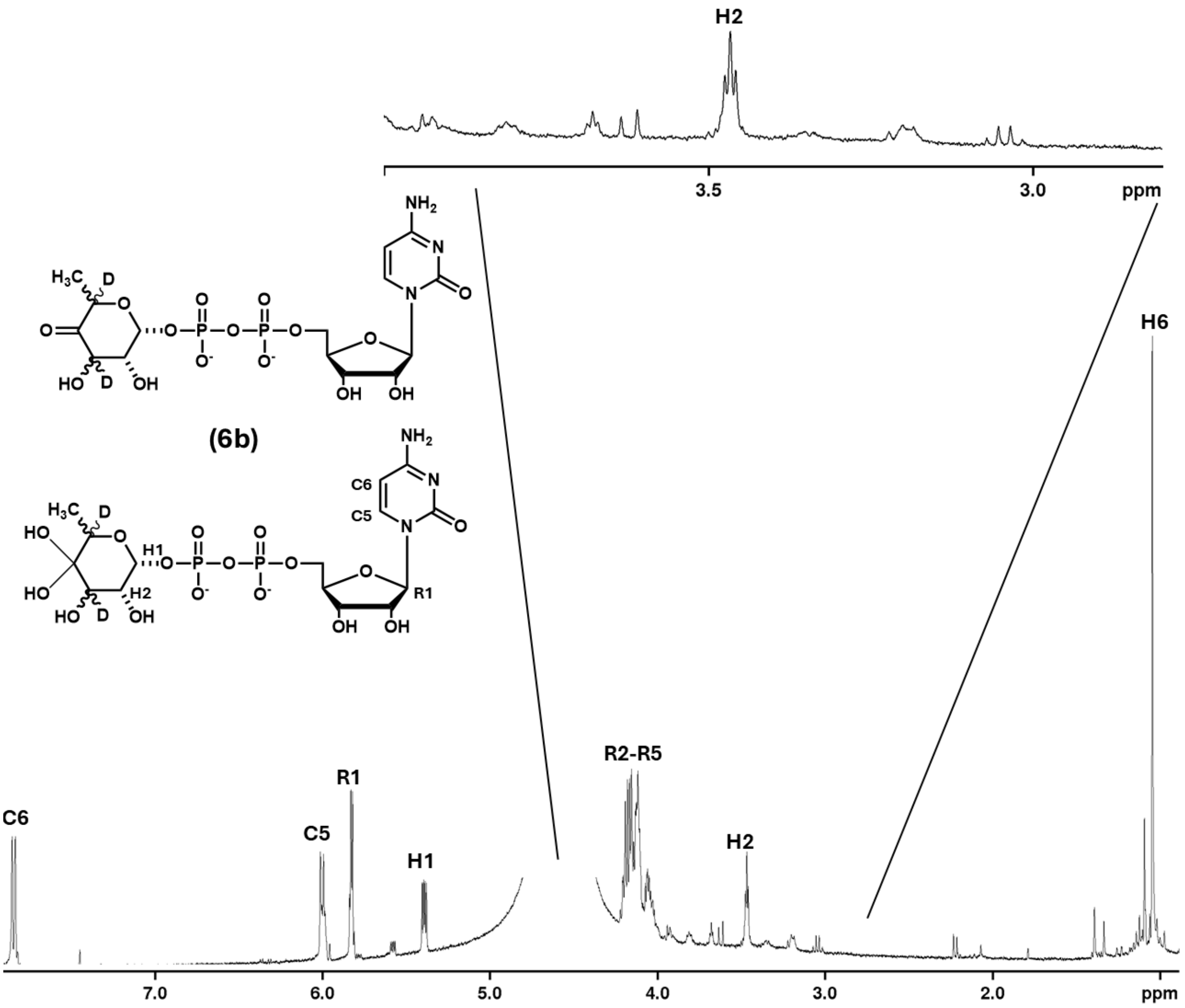

Figure S11: NMR spectrum of compound **6b** after incubation of compound **5** after incubation with the 3,5-epimerase in D_2O.

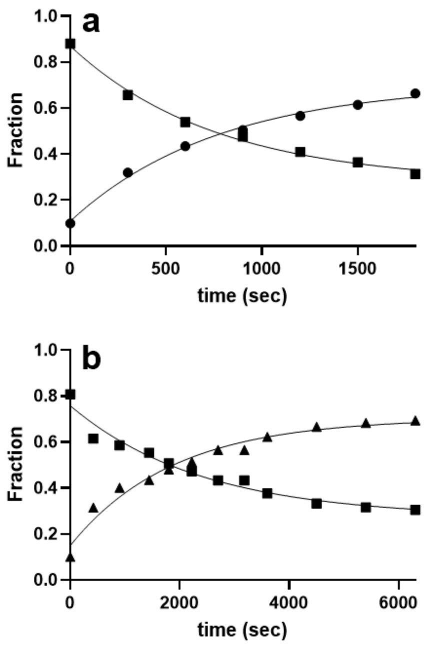

Figure S12: Time course for substrate/solvent exchange catalyzed by the 3,5 epimerase (HS:41.19) as determined by ESI-MS. (a) Exchange reaction for the conversion of 1.0 mM **5a** (filled squares) to the unlabeled product (filled circles) in 90% H_2O by the addition of 1.5 μM HS41.19. (b) Exchange reaction for the conversion of 0.5 mM **5a** (filled squares) to the dideuterated product (filled triangles) in 90% D_2O by the addition of 1.5 mM HS41.19. See **Scheme S1** for further details.

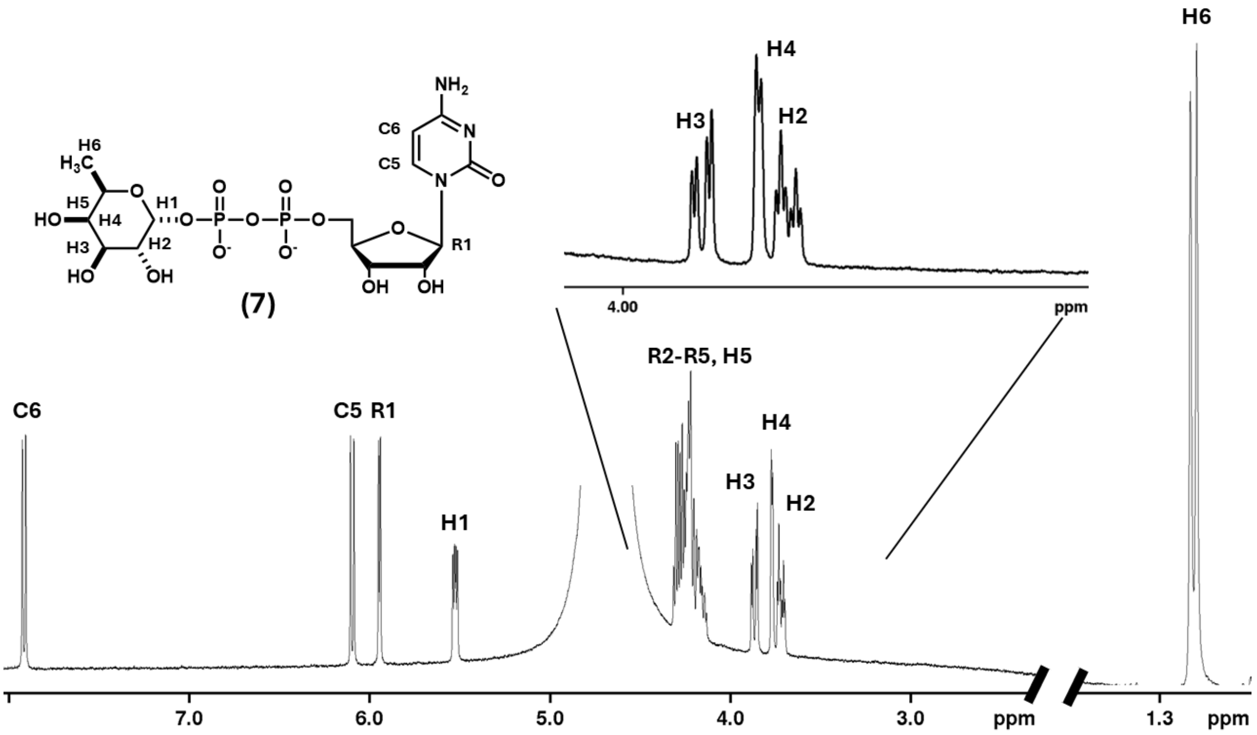

Figure S13: ^{1}H NMR spectrum of CDP-α-D-fucose (**7**). Peaks assigned with an R refer to the riboside, peaks assigned with a C refer to the cytosine protons and peaks assigned with an H refer to the fucopyranose.

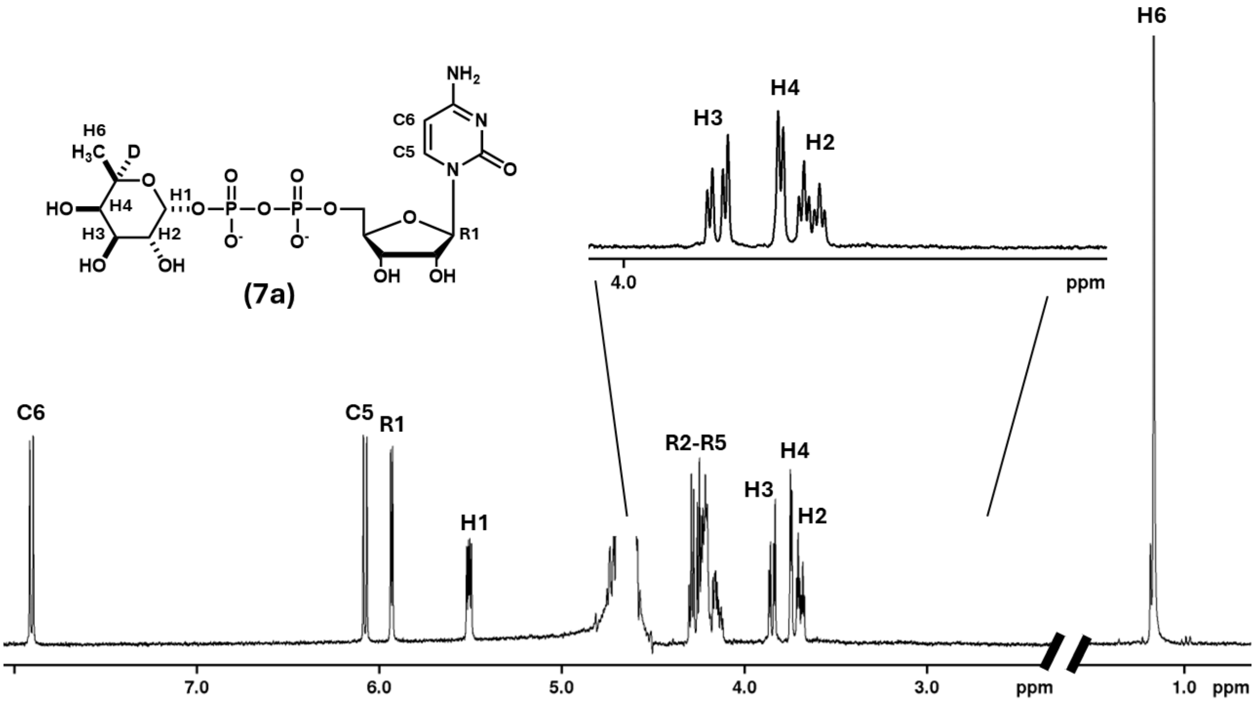

Figure S14: ^{1}H NMR spectrum of CDP-α-D-fucose (**7a**) with deuterium incorporation at C5. Peaks assigned with an H refer to the fucopyranose, peaks assigned with a C refer to the cytosine nucleotide base and peaks assigned with an R refer to the riboside.

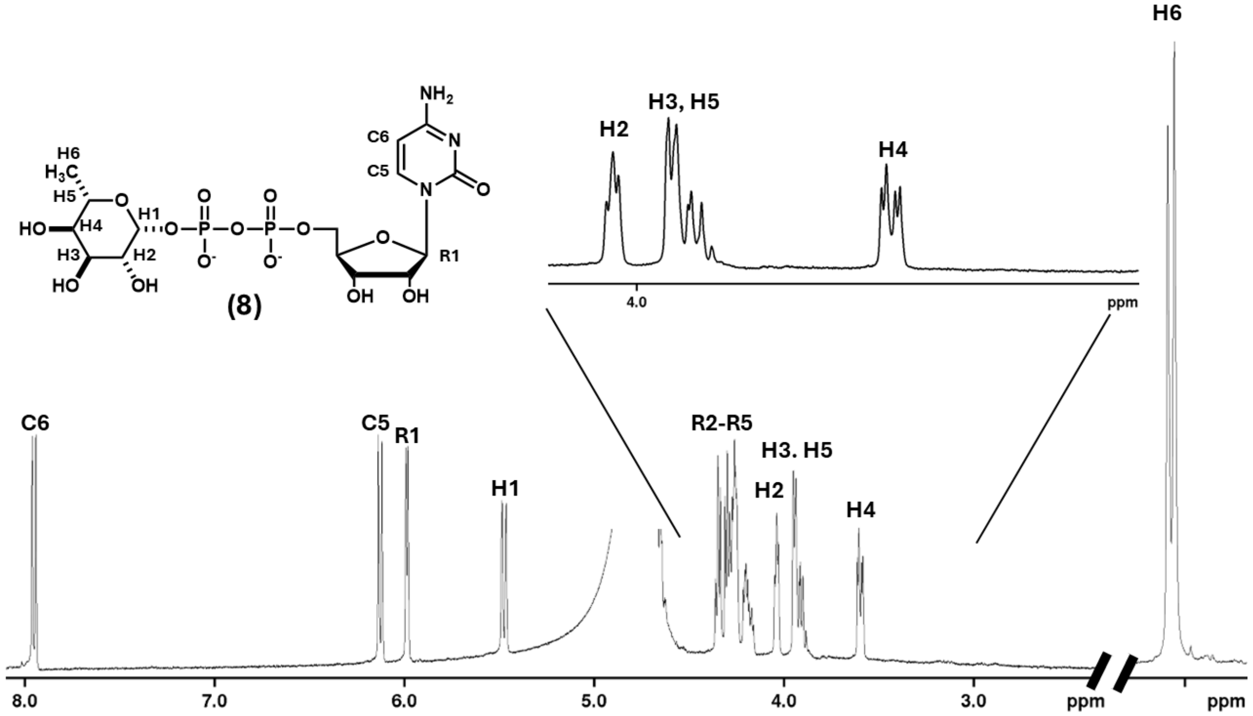

Figure S15: ^{1}H NMR spectrum of CDP-β-L-6-deoxy-altrose (**8**). Peaks assigned with a C refer to the cytosine base, peaks assigned with an R refer to the ribose sugar and peaks assigned with an H refer to the 6-deoxy-altropyranose.

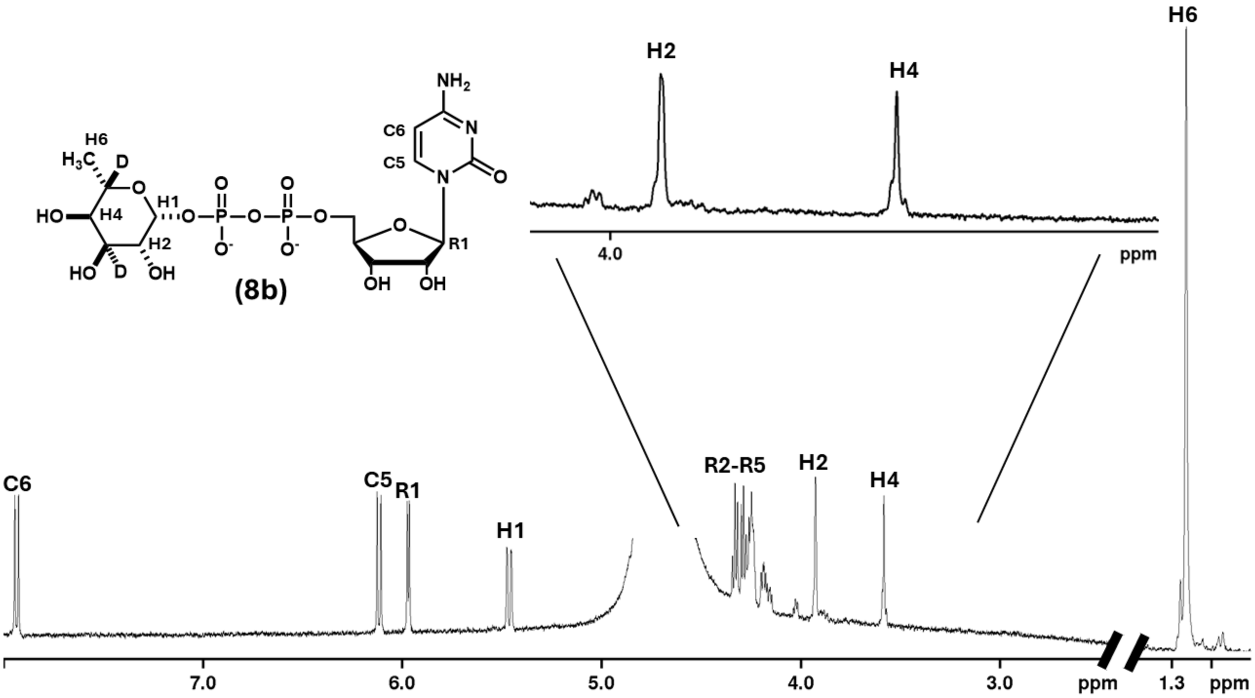

Figure S16: ^{1}H NMR spectrum of CDP-β-L-6-deoxy-altrose (**8b**) with a double deuterium incorporation at C3 and C5. Chemical shifts assigned with an H refer to the 6-deoxy-altropyranose, shifts assigned with an R refer to the riboside and shifts assigned with a C refer to the protons of the cytosine base.

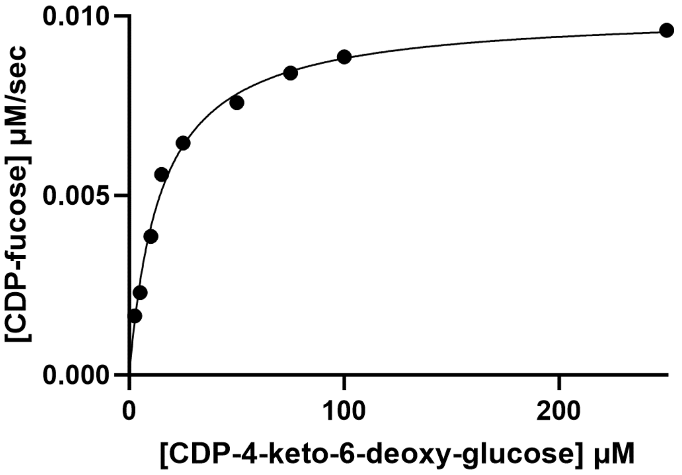

Figure S17: Michaelis-Menten plot of HS:41.18 activity using UV-vis spectroscopy. Reactions were performed in HEPES buffer (pH 7.5) with 0.16 µM NADPH, 45 nM reductase and CDP-α-D-glucose that had been dehydrated by HS:41.20.

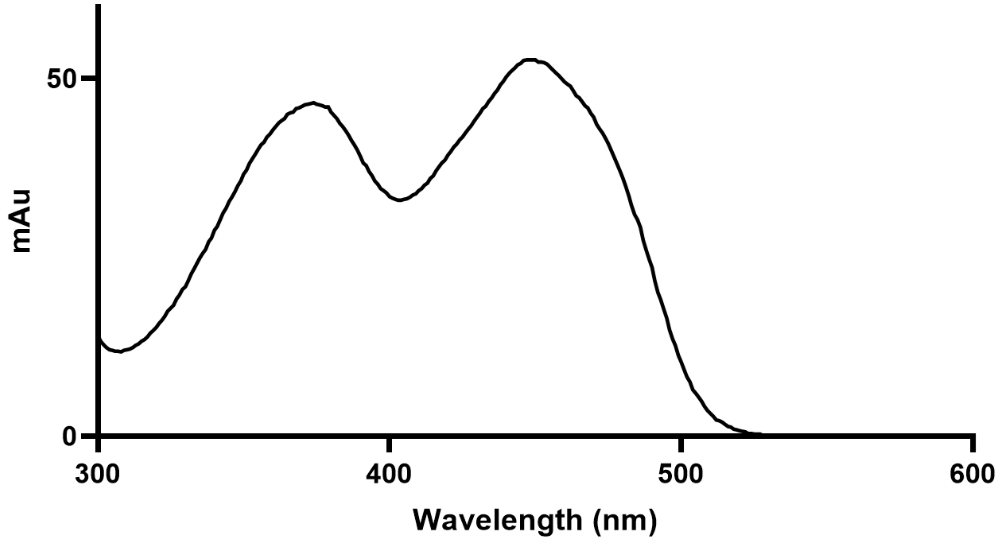

Figure S18: UV-Vis spectrum of the CDP-α-D-fucopyranose mutase (HS:41.17) after heat denaturation and centrifugation.

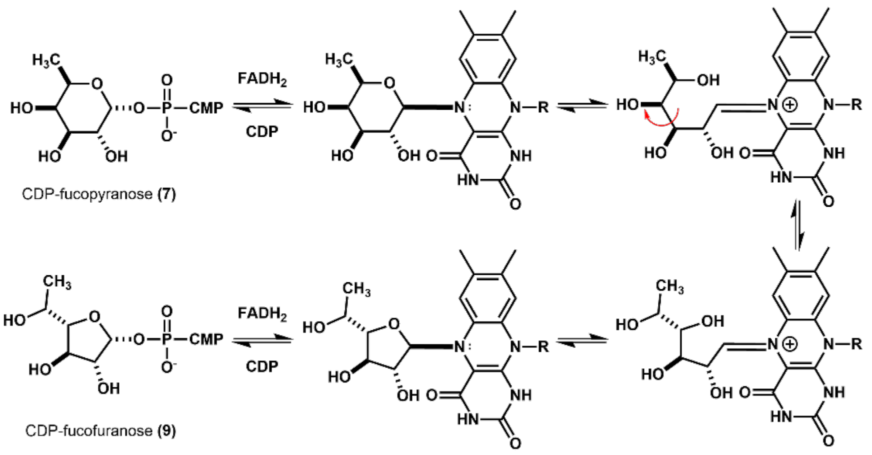

Figure S19: Proposed chemical mechanism of HS:41.17 CDP-fucopyranose mutase. The N5 of $FADH_2$ performs nucleophilic attack on C1 of CDP-fucose and releases CDP. The next step forms an iminium ion which cleaves the pyranose ring and enables recombination with the hydroxyl group at C4.

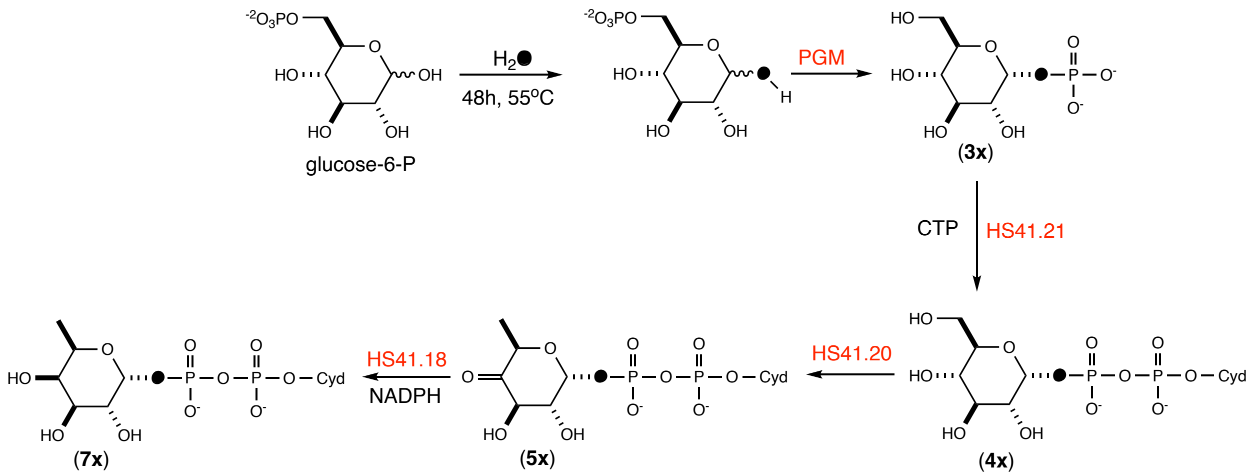

Figure S20: Chemoenzymatic synthesis of [1-^{18}O]-CDP-D-fucose (**7x**).

Chemoenzymatic synthesis of [1-^{18}O]-CDP-D-fucose (7x). A 1.0 mL solution of 85 mM glucose-6-phosphate in 97% oxygen-18 labeled water was incubated for 48 h at 55 ^{o}C to exchange the oxygen at C1 with the labeled oxygen from the solvent. In a volume of 3.0 mL, 8.5 mM of the oxygen-18 labeled glucose-6-phosphate was incubated at 25 ^{o}C for 18 h with 10 mM CTP, 7.0 mM $MgCl_2$, 15 μM phosphoglucomutase (PGM), 0.5 μM HS41.21, 60 nM pyrophosphatase and 50 mM HEPES, pH 8.5, to make [1-^{18}O]-CDP-D-glucose (**4x**) through the *in situ* formation of [1-^{18}O]-glucose-1-phosphate (**3x**). The product (**4x**) was applied to a 5 mL Hi-Trap anion exchange column and eluted with a 0-500 mM gradient of NH_4HCO_3. ESI-MS analysis of the purified material indicated an oxygen-18 incorporation of 96%. A 0.5 mL solution of [1-^{18}O]-CDP-D-glucose (**4x**) was dehydrated to compound **5x** and then reduced to [1-^{18}O]-CDP-D-fucose (**7x)** in 50 mM P_i buffer, pD 7.5, containing 0.5 μM HS41.20, 6.0 μM HS41.18 and 12 mM NADPH. The reactions were allowed to proceed for 18 h. The product (**7x**) was purified using a Carbopac Dionex PA1 column and elution with a linear gradient of 2.0 M ammonium acetate, pH 7.5. Fractions containing the [1-^{18}O]-CDP-D-fucose (**7x**) were pooled, lyophilized and reconstituted in 50 mM phosphate buffer in D_2O. The yield was 2.0 μmoles of compound **7x** for use in the positional isotope exchange reaction catalyzed by pyranose/furanose mutase, HS41.17. The reaction scheme is illustrated in **Figure S20**.

Synthesis of CDP-α-D-fucofuranose (9).

The synthesis CDP-α-D-fucofuranose (**9**) consists of two parts: preparation of α-D-fucofuranose-1-phosphate (**S7**) and the preparation of CDP-α-D-fucofuranose (**9**).

Synthesis of α-D-fucofuranose-1-phosphate (**S7**). D-Fucose (**S1**) was converted by acid-catalyzed methylation (AcCl/MeOH) into a mixture of the α- and β-isomers of furanose and pyranose forms (**S2**), (methyl-β-fucofuranose was the predominant component). Crude **S2** was acetylated in a mixture of Ac_2O/AcOH containing 0.2% sulfuric acid. Product **S3** (β-isomer) was isolated by silica gel column chromatography (hexanes:EtOAc, 5:2). **S3** was converted into **S4** using 33% HBr/AcOH in dichloromethane. Without isolation, **S4** was phosphorylated in anhydrous toluene with an excess of triethylammonium dibenzyl phosphate. The fully protected α-D-fucofuranosyl-1-phosphate (**S5**) was isolated by silica gel chromatography in a 40% yield. Catalytic hydrogenation of **S5**, using Pd/C catalyst (10%) in ethyl acetate/ethanol/triethylamine (1:1.4:0.8) mixture, followed by deacetylation in methanol/water/triethylamine (3:1.5:1.5) afforded α-D-fucofuranose-1-phosphate bis-triethylammonium salt (**S7**).[1]

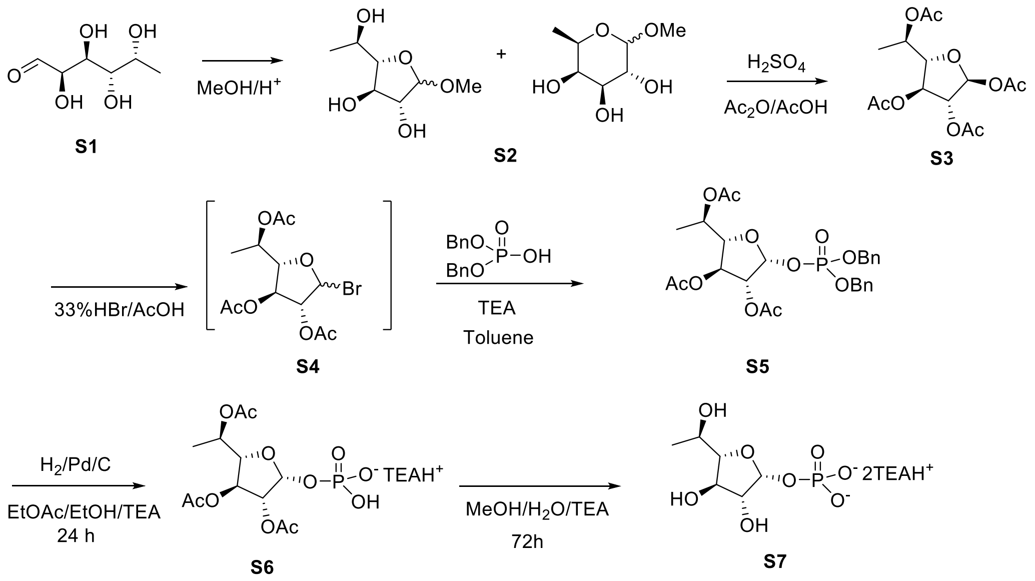

Scheme S2: Scheme for the synthesis of intermediate **S7**.

Synthesis of CDP-α-D-fucofuranose (9). CDP-α-D-fucofuranose (**9**) was synthesized by coupling of α-D-fucofuranose-1-phosphate bis-triethylammonium salt **S7** (1 equiv) with cytidine 5'-phosphoromorpholidate dicyclohexylcarboxamidine salt **S8** (2.0 equiv) using the 4,5-dicyanoimidazole (DCI) (2.5 equiv) promoted coupling procedure at 35 °C for 30 h (**Scheme S3**).[2]

To a solution of cytidine-5'-phosphoromorpholidate (46 mg, 0.067 mmol, 2 equiv) and α-D-fucofuranose-1-phosphate bis-triethylammonium salt (30 mg, 0.034 mmol, 1 equiv) in anhydrous DMF (0.5 mL) was added 4,5-dicyanoimidazole (DCI) (9.8 mg, 0.083 mmol, 2.5 equiv). The reaction was stirred at 30 °C for 30 h. The solution was concentrated *in vacuo*. The residue was extracted with deionized water (3 x 1 mL), and the combined adqueous solution was frozen and stored at -80 °C. Product **9** was purified using a 5 mL HiTrap Q anion exchange column using a linear gradient of 0 - 100%, 220 mM TEAB buffer, pH 7.5. Product **9** was further purified using a Carbopac Dionex PA1 column (2M ammonium acetate buffer, pH 7.1). Fractions containing the desired product were collected and lyophilized to obtain 3 mg (16%) of **9**. The ^{1}H NMR spectrum is provided in **Figure S21** and the ^{31}P NMR spectrum is provided in **Figure 22**.

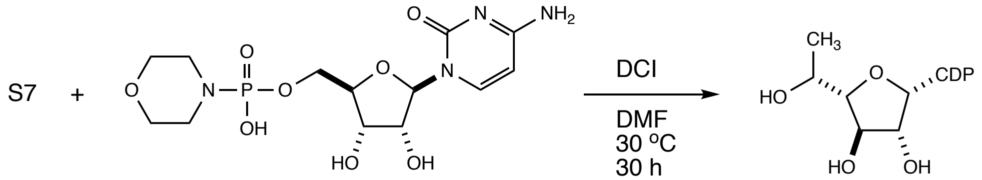

(**S8**)

(**9**)

Scheme S3: Chemical synthesis of compound **9**.

^{1}H NMR (400 MHz, D_2O) δ 8.05 (d, J = 7.8Hz, 1H), 6.18(d, J = 7.8Hz, 1H), 5.96 (d, J = 4.0 Hz, 1H), 5.61 (dd, J_1 = 4.5 Hz, J_2 = 5.4 Hz, 1H), 4.35-4.29 (m, 2H), 4.28–4.24 (m, 2H), 4.21-4.15 (m, 1H), 4.13-4.09 (m, 1H), 4.04 (t, J = 7.0 Hz, 1H), 3.85 (quin., J = 6.7 Hz, 1H), 3.59 (t, J = 7.2 Hz, 1H), 1.16 (d, J = 6.2 Hz, 3H) ppm;

^{31}P NMR (162 MHz, D_2O) δ −11.3 (d, *J* = 21.0 Hz, 1P), −12.7 (d, *J* = 21.0 Hz, 1P) ppm.

(ESI$^-$) *m/z* $[M - H]^-$ calcd. for $C_{15}H_{24}N_3O_{15}P_2$: 548.0677, found: 548.0686.

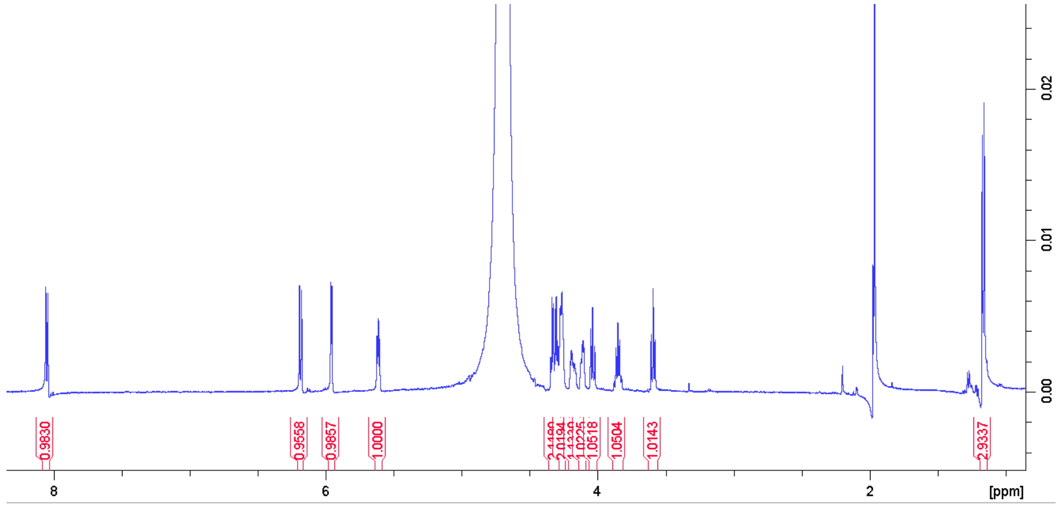

Figure S21: ^{1}H NMR spectrum of chemically synthesized compound **9.** The resonance a ~2.0 ppm is from acetate.

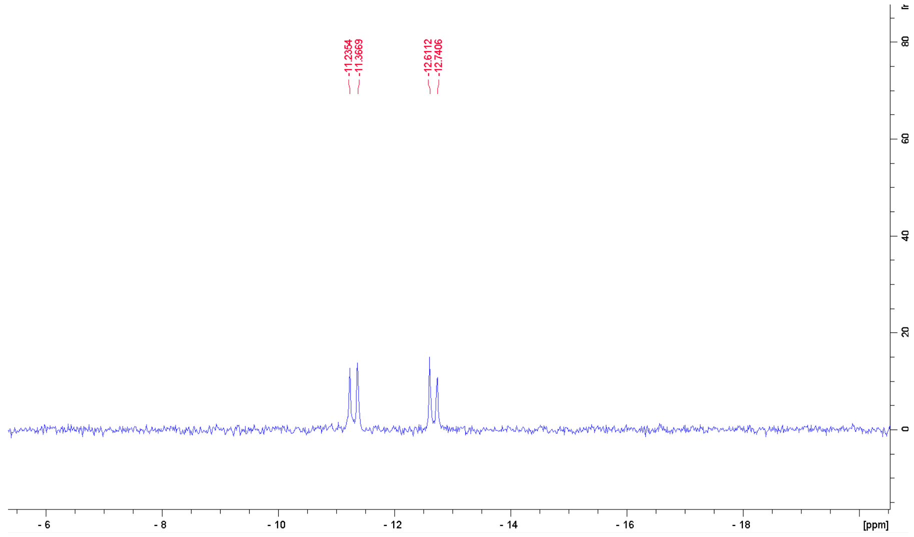

Figure S22: ^{31}P NMR spectrum of chemically synthesized compound **9**.

Synthesis of CDP-6-deoxy-β-L-altrofuranose (**10**). Synthesis CDP-6-deoxy-β-L-altrofuranose (**10**) consists of two parts: preparation of 6-deoxy-β-L-altrofuranose-1-phosphate **S13** (**Scheme S4**) and preparation of CDP-6-deoxy-β-L-altrofuranose (**10)** (**Scheme S5**). The procedures for the reactions are described in the literature.[1, 2, 3]

Synthesis of 6-deoxy-β-L-altrofuranose-1-phosphate (S13): D-Galactose (**S8**) was converted into the 6-deoxy-L-altrofuranose derivative (**S9**) according to previously described procedures.[3] Acetylated derivative (**S11**) was obtained in two steps and converted into the fully protected 6-deoxy- β-L-altrofuranose-1-phosphate (**S12**) by the procedures described earlier (yield 35%)**.**[1] Catalytic hydrogenation of **S12**, applying Pd/C catalyst (10%) in ethylacetate/ethanol/triethylamine (1:1.4:0.8) mixture, followed by deactelylation in methanol/water/triethylamine (3:1.5:1.5) afforded 6-deoxy-β-L-altrofuranose-1-phosphate bistriethylammonium salt (**S13**)**.**

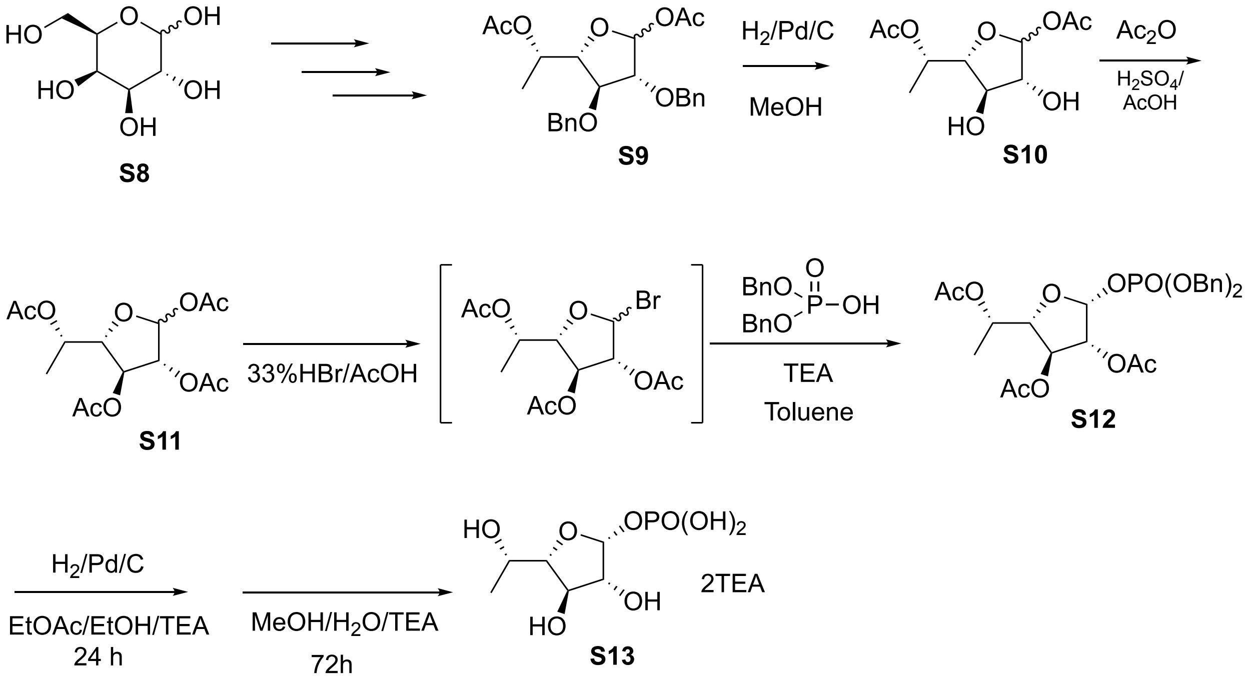

Scheme S4: Synthetic scheme for the preparation of intermediate compound **S13**.

Synthesis of 10: Synthesis and purification of CDP-6-deoxy-β-L-altrofuranose (**10**) was identical to that described for CDP-α-D-fucofuranose (**9**).[2]

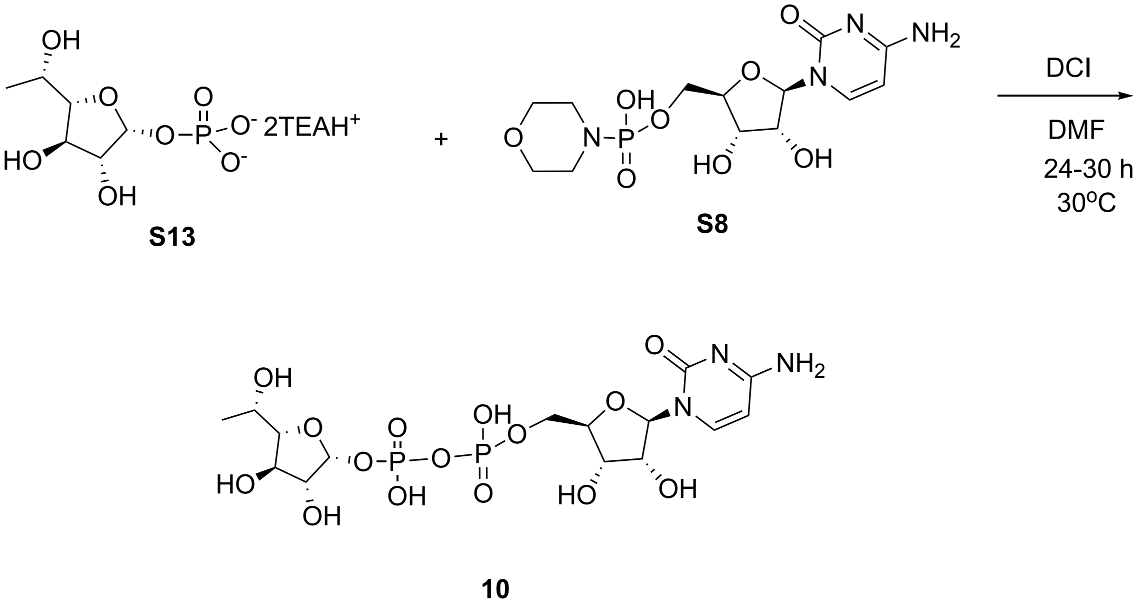

Scheme S5: Scheme for the chemical synthesis of compound **10.**

CDP-6-deoxy-β-L-altrofuranose (10):

^{1}H NMR (400 MHz, D_2O) δ 8.07 (d, J = 8.4 Hz, 1H), 6.20 (d, J = 8.0 Hz, 1H), 5.96 (d, J = 4.0 Hz, 1H), 5.60 (t, *J* = 4.8 Hz, 1H), 4.37 -4.29 (m, 2H), 4.28–4.21 (m, 3H), 4.20-4.09 (m, 3H), 4.02-3.96 (m, 1H), 3.75 (dd, J_1 = 7.2 Hz, J_2 = 4.2 Hz, 1H), 1.18 (d, J = 6.5 Hz, 3H) ppm;

^{31}P NMR (162 MHz, D_2O) δ −11.2 (d, *J* = 21.0 Hz, 1P), −12.6 (d, *J* = 21.0 Hz, 1P) ppm.

(ESI$^-$) m/z [M - H]$^-$ calcd. for $C_{15}H_{24}N_3O_{15}P_2$: 548.0677, found: 548.0692.

References

1. Errickson Simons, M., Narindoshvili, T., Raushel, F. M., Biosynthesis of UDP-β-L-Arabinofuranoside for the Capsular Polysaccharides of *Campylobacter jejuni*. *Biochemistry*, **2023**, *62, 3012-3019.*
2. Chen, W.-J., Han, Sh-B., Xie, Zh-B., Huang, H-Sh., Jiang, D-H., Gong, Sh-Sh., Sun. Q. Efficient Synthesis of UDP-Furanoses via 4,5-Dicyanoimidazole(DCI)-Promoted Coupling of Furanosyl-1-Phosphates with Uridine Phosphoropiperidate. *Molecules*, **2019**, *24, 655.*
3. Ting, Y.-J., Yang, Y.-H. , Ho, G.-M. , Hung, ShCh. Synthesis of rare L-altro sugars and C6-Derivatives. *Tetrahedron*, **2023**, *143, 133555*.
